# Advanced cell-derived drug delivery systems for pulmonary diseases: from bench to bedside

**DOI:** 10.1080/10717544.2025.2564814

**Published:** 2025-09-30

**Authors:** Fei Li, Wenjie Xu, Junyong Wu, Jiwen Zhang, Xingyu Wei, Dehua Liao

**Affiliations:** ^a^ Department of Pharmacy, Hunan Cancer Hospital, the Affiliated Cancer Hospital of Xiangya School of Medicine, Central South University, Changsha, China; ^b^ School of Pharmacy, University of South China, Hengyang, China; ^c^ Department of Pharmacy, The Second Xiangya Hospital, Central South University, Changsha, China

**Keywords:** Cell-derived carrier, Extracellular vesicle, Pulmonary diseases, Drug delivery, Translational medicine

## Abstract

Nowadays, pulmonary diseases (PDs) are among the leading causes of mortality worldwide. Conventional therapeutic approaches exhibit disappointing efficacies due to difficulty in drug delivery and systemic cytotoxicity. In recent years, novel formulations of therapeutic drugs rised as alternatives for clinical treatment. Among them, cell-derived drug delivery systems (CDDSs) have garnered attention for their potential in treating PDs. By harnessing the innate migratory capabilities, barrier-crossing potential, high biocompatibility, and substantial drug-loading capacity of cell derivatives, CDDSs offer a promising approach for PD treatment. However, there was no systemic report in summarizing CDDSs in PDs. In this review, We first examined the biological properties and therapeutic advantages of various CDDSs in the context of PDs, including red blood cells (RBCs), stem cells, platelets, macrophages, neutrophils, tumor cells, microalgae, extracellular vesicles (EVs) and biomimetic cell membrane. We then discussed common preparation strategies and different modification methods of CDDSs. Finally, we summarized the current therapeutic advancements of CDDSs in multiple PDs. We hope this review serves as a valuable reference for utilizing CDDSs in the treatment of PDs and other diseases.

## Graphical abstract

**Figure:**
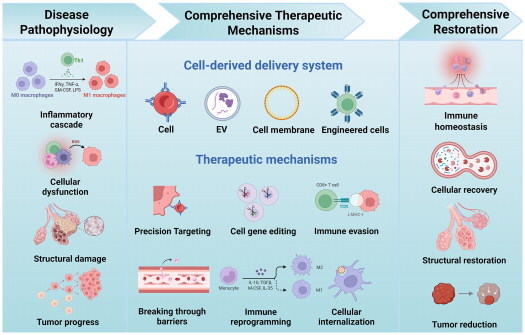

## 1. Introduction

Pulmonary diseases (PDs) are the third leading cause of morbidity and mortality worldwide, after cancer and cardiovascular diseases (Geiger et al. 2017). PDs encompass a series of conditions including pulmonary infection, acute lung injury (ALI), lung cancer, asthma and pulmonary fibrosis (PF), etc. Collectively, these conditions contribute to approximately 7.6 million fatalities annually (Agusti et al. 2022). Despite decades of clinical application, traditional treatments for PDs, such as intravenous pharmacotherapy, inhalation therapy, surgery, and rehabilitation continue to face significant challenges (Amaral et al. 2023; Ferrera et al. 2021). For example, the off-target effect of therapeutics, the occurrence of bacteria resistance, the impediment of physiological barriers in the alveoli and airways and inherent surgical risks (Mukherjee and Bhatt 2022; Cillóniz et al. 2021; Wan et al. 2023). These limitations underscore an urgent and unmet demand for innovative therapeutic strategies in PDs management.

Nanotechnology, as an emerging filed, has found extensive application in PDs. This is attributed to its capacity to improve medication bioavailability and targeting, increase the period of drug accumulation in the lungs, and decrease systemic adverse effects (Zhong et al. 2021; Doroudian et al. 2019). For instance, Paclitaxel (PTX), a chemotherapeutic medication used to treat lung cancer, is limited in clinical use due to its low water solubility, low bioavailability, and severe toxicity (Landesman-Milo et al. 2015). However, nanoformulations of PTX have received clinical approval and have shown improved therapeutic efficacy while reducing toxicity. Furthermore, nanoparticles can be engineered with surface modifications to facilitate targeted therapy, and in lung cancer therapy, nanoparticles can be made to selectively attach to receptors on the surface of tumor cells, increasing the drug’s ability to target tumor sites and reducing negative effects on healthy tissues (Chan et al. 2020). Despite these advances, the application of conventional nanodelivery systems in PDs encounters critical hurdles: the aggressive clearance of nanoparticles by the mononuclear phagocyte system drastically curtails their therapeutic efficacy (Dobrovolskaia and McNeil 2007). Furthermore, certain nanomaterials interact adversely with respiratory tissues. More specifically, these interactions might result in the generation of free radicals, which alveolar macrophages may then collect. The accumulation causes an inflammatory reaction that can cause severe inflammation and PD development (Thu et al. 2023). In recent years, live cells, extracellular vesicles(EVs), exosomes, and biomimetic cell membranes have emerged as carriers in the field of PD treatment, demonstrating advantages that traditional treatments and nanoparticle treatments cannot match. Live cells possess excellent biocompatibility, low immunogenicity, prolonged circulation time, tissue targeting capability, and the ability to traverse biological barriers (Liu et al., 2024; Yang et al. 2022). For instance, mesenchymal stem cells (MSCs) exhibit natural targeting properties, actively homing to damaged lung tissue while maintaining low immunogenicity and high safety profiles (Cheng et al. 2022; Han et al. 2023). Evs are nanoscale vesicles actively secreted by cells. Exosomes, as an important member of this family, have a diameter ranging from 30 to 150 nanometers and contain proteins, messenger RNA, lipids, and various active substances such as cytokines. Exosomes exhibit excellent biocompatibility and low immunogenicity, enabling them to cross multiple biological barriers and achieve efficient cellular internalization (Wang et al. 2022; Sharma et al. 2021; H. Wang et al. 2021; Elsharkasy et al. 2020; Li et al., 2021; Xu et al. 2024). Bionic cell membranes retain the surface receptors and functional proteins of their source cells, giving bionic nanocarriers advantages such as high targeting, long circulation time, good biocompatibility, and strong immune evasion capabilities. For example, red blood cell membrane-coated nanoparticles have good biocompatibility and long circulation characteristics (Nguyen et al. 2023). In summary, cell, EVs, exosomes, and biomimetic cell membranes as carriers have demonstrated unique advantages in the treatment of PDs, bringing new hope for the treatment of PDs. In recent years, although there have been many reviews on the use of cells, EVs, exosomes, and biomimetic cell membranes for drug delivery (Yang et al. 2022; Batrakova and Kim 2015; He et al. 2020), there is still relatively little research on the treatment of PDs. Therefore, in this review, we aim to fill this gap by analyzing the latest advances in the treatment of PDs using cells, EVs, exosomes, and biomimetic cell membranes. We will discuss their biological functions, advantages and limitations as drug delivery carriers, and potential applications and clinical translation status (Table 1). It is our hope that this comprehensive analysis will serve as a valuable reference for the development of innovative drug delivery systems (DDSs) for clinical PD therapy, thereby promoting the application of more effective cell-derived formulations and facilitating large-scale production in the future.

**Table 1.** Advantages, limitations, and clinical translation readiness of three delivery systems.

	Cell delivery system	Extracellular vesicle delivery system	Bionic cell membrane delivery system
Function of Cells from Different Sources	MSCs: Modulates immunity and promotes repair. Suitable for inflammation/fibrosis (Xie et al. 2021). Macrophages: Naturally pro-inflammatory/tumor-targeting (Guo and Qian 2024). Used for treating inflammation/tumors. Neutrophils: Inflammation-targeting (Wang et al. 2021). T cells: Highly specific targeting (CAR-T). Tumor immunotherapy (Larson and Maus 2021).	MSCs-EVs: Modulate immunity/promote repair (Rezaie et al. 2022). Macrophage-EVs: Target inflammation/tumor microenvironment (de Carvalho et al. 2023). Tumor cell-EVs: Target homologous tumors (Rabe et al. 2021). Neutrophil-EVs: Target inflammatory sites, penetrate pulmonary barriers (Thomas et al. 2024).	Red blood cell membrane: Immune evasion, suitable for extended circulation time (Xia et al. 2019). Macrophage membrane: Inflammation/tumor targeting, immune evasion (Wu et al. 2022). Stem cell/tumor cell membrane: Tumor targeting (He et al. 2020).
Advantages	(1) Intrinsic biological activity: Secretes therapeutic factors and participates in repair (e.g. MSCs/immune cells); (2) Superior targeting: Endogenous migration (e.g. immune cells chemotaxis toward inflammation and tumors); (3) High biocompatibility; (4) Barrier-crossing capability.	(1) Excellent biocompatibility and low immunogenicity; (2) Natural ability to traverse biological barriers; (3) Inherent biological function: Carries progenitor cell signaling molecules (nucleic acids, proteins, lipids) to regulate target cells; (4) Nanoscale size (approximately 30–150 nm): Facilitates penetration into lung tissue/deep delivery; (5) Natural platform as a drug/molecular carrier: Capable of loading small molecules, nucleic acids, and proteins; (6) Higher safety potential than living cells: No risk of division or tumorigenicity.	(1) Excellent biocompatibility; (2) Strong platform compatibility: Capable of encapsulating diverse core nanoparticles (polymers, liposomes, metal nanoparticles), significantly enhancing their performance; (3) High engineering potential: Surface functionalization with molecules (ligands, antibodies, PEG); (4) Strong immune evasion capability: Evades phagocytic clearance in the bloodstream; (5) Mitigates risks associated with living cell therapies: No metabolic activity/proliferative capacity.
Limitations	(1) Extremely high production and scaling costs: Cell culture expansion and stringent QC; (2) Batch-to-batch variability: Influenced by donor differences and activation status; (3) Drug loading efficiency and controlled release challenges: Non-specialized carriers, limited loading capacity, and difficult release control; (4) Significant safety concerns: Tumorigenic/mutagenic risks; (5) Immune response; (6) Difficulties in regulating cell fate and in vivo behavior.	(1) Low yield, large-scale production challenges, and difficulties in purification: Poor reproducibility. Not suitable for high-throughput drug manufacturing; (2) Severe lack of separation, purification, and characterization standards; (3) Insufficient endogenous targeting: Often requires additional engineered modifications to enhance targeting.	(1) Lack of living cell function: No intrinsic biological activity (secretion of factors, migration); therapy relies solely on drug delivery/membrane biomimetic properties; (2) Complex membrane extraction and coating processes: Issues include compromised membrane integrity, loss of functional proteins, impurities, and coating efficiency; (3) Difficult batch-to-batch consistency control: Variables introduced by cell source differences, membrane extraction, and coating processes; (4) Challenges in in vitro/in vivo stability and long-term storage: Relatively fragile membrane structure.
Preparation for Conversion	Some have entered Phase II/III clinical trials. (e.g. lung cancer (NCT06006390 (Chimeric Antigen Receptor T Lymphocytes \(CAR-T\)., 2023)), ARDS (NCT04371393 (Mesenchymal Stromal Cells for the Treatment of Moderate to Severe COVID-1919., 2020))).	Some have entered Phase II/III trials. (e.g. COVID (NCT05125562 (Bone Marrow Mesenchymal Stem Cell Derived Extracellular Vesicles Infusion Treatment for Mild-to-Moderate COVID-19: A Phase II Clinical Trial 2021)), ARDS (NCT05354141 (Bone Marrow Mesenchymal Stem Cell Derived Extracellular Vesicles for Hospitalized Patients With Moderate-to-Severe ARDS. A Phase III Clinical Trial 2022))).	Preclinical optimization phase.

## 2. The introduction of cell therapy

### 2.1. Biological classifications and functions of CDDSs

**Figure 1.**
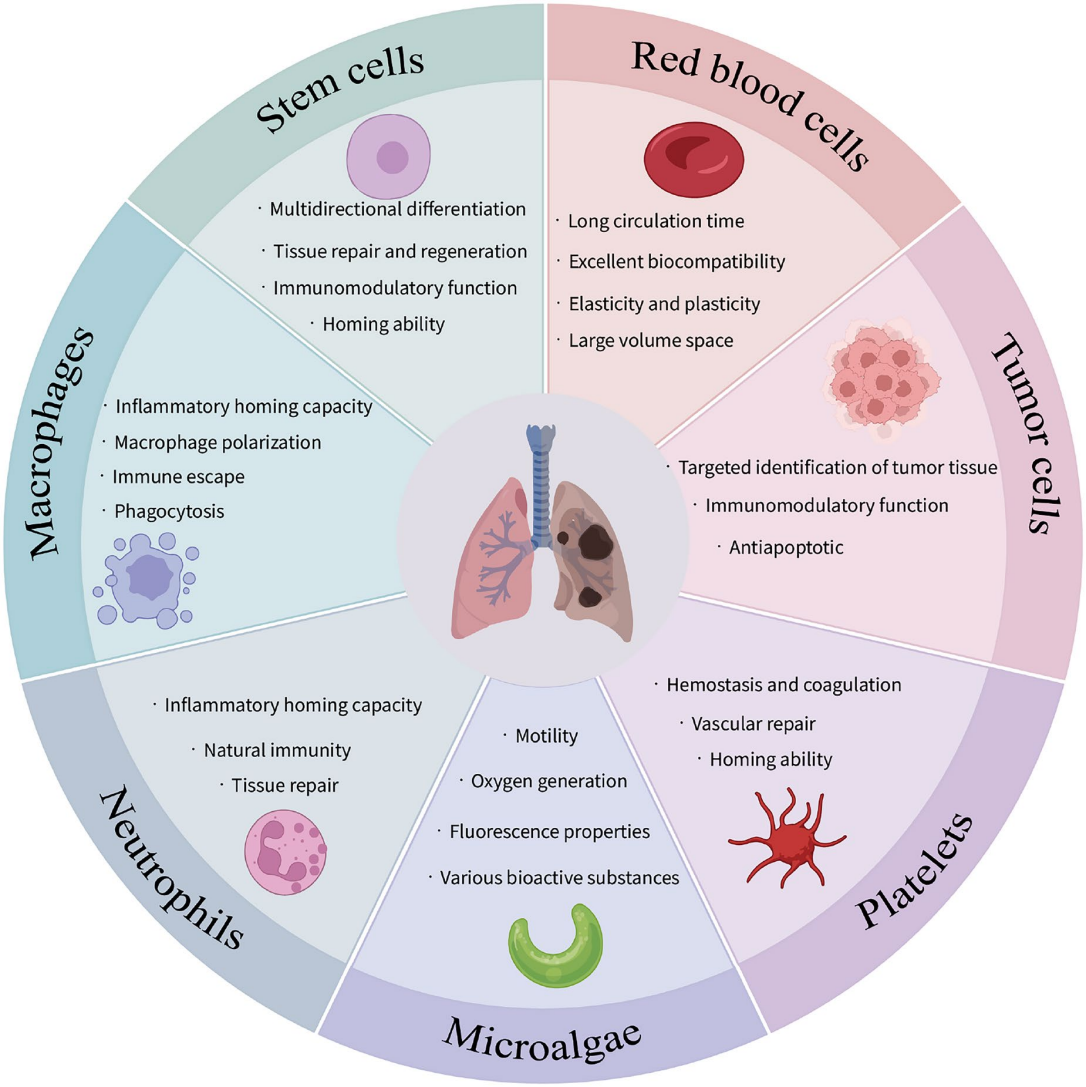
Functions of different cells in the treatment of PDs. Figure was created with BioRender.com.

RBCs, stem cells, macrophages, neutrophils, lymphocytes and others possess unique characteristics within bodies and show broad prospects in drug delivery. Here in this section, we describe the classification of various types of cells and their functions in PDs treatment (Figure 1), aiming to provide suitable drug carrier alternatives for different PDs.

#### 2.1.1. RBCs

RBCs are the most abundant type of blood cells, and their primary biological function is to transfer oxygen and carbon dioxide to sustain normal physiological metabolism (Han et al. 2018). Compared with other cells, RBCs lack nuclei and organelles in their structure and it has a biconcave structure, increasing the surface area and deformation ability of RBCs. Meanwhile, RBCs serve as carriers for transporting oxygen, which needs to be transported to all parts of the body, so it has good biocompatibility and prolonged circulation time (Villa et al. 2015; Della Pelle and Kostevšek 2021). CD47, a transmembrane glycoprotein found on the surface of RBCs, interacts with signal regulatory protein α (SIRPα) on macrophages, preventing the premature clearance of normal RBCs (Olsson et al. 2006). Moreover, RBCs’ excellent adaptability and plasticity enable them to preserve their structural integrity while moving through capillaries under mechanical stress (Li et al., 2021). Owing to their extended blood circulation time, high payload capacity, excellent biocompatibility, and low immunogenicity (Nguyen et al. 2023; Della Pelle and Kostevšek 2021; Rong et al. 2022), RBCs are deemed as highly promising drug carriers. Through physical or chemical modifications and genetic engineering, we can enhance the drug loading efficiency and targeting ability of RBCs. For instance, drugs can be incorporated into RBCs using techniques like hypotonic dialysis, chemical binding, affinity interaction, genetic engineering, or enzyme-linked reactions, all of which lead to improved drug delivery efficiency (Jiang et al. 2018; Koleva et al. 2020; Glassman et al. 2021). These features make it possible RBCs to transport and deliver medications to the lungs in an efficient manner.

#### 2.1.2. Stem cells

Stem cells are a class of undifferentiated or partially differentiated cells that have the potential to differentiate into various cell types within the body. Typically, these cells exhibit the capacity to drive tissue repair and regeneration, and they can directly target damaged lung tissue to promote its recovery (Cheng et al. 2022). Stem cells can also secrete various immune regulatory factors to alleviate inflammation and modulate local immune responses in the lung lesions (Uccelli et al. 2008). Thus, they have significant therapeutic effects on PF and inflammatory lung diseases. The primary classes of stem cells encompass embryonic stem cells (ESCs), pluripotent stem cells (PSCs), and MSCs (Kolios and Moodley 2013). Due to ethical considerations, PSCs and MSCs are commonly engineered as drug delivery carriers, while ESCs are seldom used for drug delivery. MSCs can be readily obtained from a variety of human tissues, including bone marrow, skin, blood, and adipose tissue (Khayambashi et al. 2021; Sellheyer and Krahl 2010; Hassan et al. 2019; Ahn et al. 2013), which has facilitated their widespread use in clinical settings. Their low immunogenicity nature makes them possible to reduce immune rejection when delivery exogenous drugs (Wei et al. 2013). Meanwhile, their homing ability to the active inflammatory response sites in the body enables them to be better utilized in tumor treatments (Xie et al. 2021; Wang et al. 2016). Additionally, the use of genetic engineering to modify MSCs can enhance their targeted migration toward damaged tissues and areas of inflammation (Su et al. 2021).

#### 2.1.3. Platelets

Platelets are vital to human blood circulation, and in addition to coagulation and hemostasis, they also participate in other physiological processes, including thrombosis and vascular repair (Zhu et al. 2024; Lu et al. 2019). The CD47 molecule on platelets prevented them from macrophage engulfment and thereby extending their lifespan in circulation, approximately 8–10 days (Olsson et al. 2005; Xiao et al. 2022). The natural biocompatibility minimizes the immune rejection responses against platelets-based drug delivery platforms (Kailashiya et al. 2019). These properties make them same as RBCs, suitable for long-term drug delivery. Compared to RBCs, platelets have a significant characteristic. Their innate tropism allows them to concentrate at sites of damaged tissue, tumor areas, and regions of vascular inflammation (Han et al. 2019; Han et al. 2020). Thus, platelets have the potential to serve as carriers for treating lung cancer and lung infections.

#### 2.1.4. Macrophages

Macrophages, which originate from blood monocytes, are versatile innate immune cells distributed across various tissues in the body (Guo and Qian 2024). Unlike RBCs, which have a relatively short lifespan, macrophages can survive for several months to years. This extended lifespan endows them with a longer circulation time, thereby facilitating the prolonged retention of drugs within the body (Liang et al. 2021). Macrophages demonstrate significant plasticity, differentiating into M1 and M2 macrophages, and producing a variety of cytokines that promote healing and anti-inflammatory responses (Liu et al., 2024). As a result, when medications are combined with macrophages, effective drug delivery is achieved, and by increasing their biological activity, therapeutic effects are also improved. Due of their powerful phagocytosis, macrophages are able to cytosolize chemical medicines and drug-carrying nanoparticles. As immune cells, macrophages are able to evade the mononuclear phagocytosis system’s (MPS) phagocytosis, which prevents drug breakdown and increases its stability (Liang et al. 2021). Additionally, macrophages are naturally able to migrate to inflammatory sites and are frequently found in the late phases of acute inflammation or throughout chronic inflammation (Guo and Qian 2024), which makes them perfect for drug delivery in inflammatory PDs. Also, tumor-associated macrophages (TAMs) can survive and function within tumor tissues, where they can control tumor growth and metastasis (Wróblewska et al. 2023), making TAMs a promising platform for developing DDSs targeted at treating lung cancer.

#### 2.1.5. Neutrophils

An essential part of the innate immune system, neutrophils are the most prevalent form of white blood cells in the human circulation. They take part in defense against cancers, autoimmune reactions, tissue healing after acute injury, and the regulation of chronic inflammatory responses, among other vital functions (Liew and Kubes 2019). Neutrophils can act as medication carriers to target lung inflammation because they can migrate to inflammatory areas in response to inflammatory signals (H. Wang et al. 2021). The effectiveness of medicine delivery is increased when neutrophils are used as carriers, especially when treating ARDS and acute lung damage (Chu et al. 2018). In addition to serving as drug delivery systems, neutrophils also help treat diseases by influencing immune responses. Neutrophil extracellular traps (NETs)-DNA, for example, can cause NF-κB-dependent autoimmune reactions in chronic obstructive pulmonary disease (COPD) through the cyclic GMP-AMP synthase/toll-like receptor 9 (cGAS/TLR9) pathway. COPD symptoms can be alleviated by preventing the formation of NETs (Chen et al. 2024).

#### 2.1.6. Tumor cells

As a class of cells that proliferate abnormally, tumor cells have biological roles such as promoting angiogenesis, evading apoptosis, promoting rapidly cell division, facilitating migration and invasion, and having unique metabolic pathways (Lei et al. 2020). Because of their particular biological properties, tumor cells have a number of particular advantages when it comes to drug delivery. During therapy, adverse effects and immunological reactions might be considerably decreased by their biocompatibility and biodegradability. Furthermore, when used as drug carriers, tumor cells’ resistance to apoptosis allows them to avoid immune system clearance, prolonging the duration of drug circulation in the body (Du et al. 2021). Additionally, tumor cells exhibit the capacity to alter immunological responses, either activating or inhibiting them. This allows them to play an immunomodulatory role in the therapy of PDs (Guo et al. 2019). Finally, because tumor cells can identify homologous cells, they can adhere to and target tumor tissues specifically, raising the medication concentration at the tumor site (Harris et al. 2019; Vijayan et al. 2018). The unique characteristics of tumor cells as drug carriers highlight their considerable potential for PD treatment. They enhance drug targeting, biocompatibility, and delivery efficiency, while simultaneously minimizing systemic side effects and providing immunomodulatory benefits. Through modern genetic engineering techniques, we also can engineer tumor cells to carry therapeutic molecules, such as drugs, antibodies, or specific RNAs, to achieve precise drug delivery (Liu et al., 2024; Xu et al. 2019). However, The use of tumor cells as carriers for the treatment of PDs also faces numerous challenges, with safety and immune clearance rates being particularly prominent issues. Tumor cells inherently possess abnormal biological characteristics, such as uncontrolled proliferation and metastasis capabilities. When tumor cells are engineered as therapeutic carriers, it is essential to ensure that their tumorigenicity is completely eliminated or effectively controlled; otherwise, they may induce new tumors in patients. At the same time, although tumor cells originate from the body itself, they undergo significant genetic and phenotypic changes, resulting in characteristics that are completely different from those of normal cells. These abnormal characteristics cause the immune system to regard them as’foreigners,’making them easy for immune cells to quickly identify and activate clearance mechanisms (Vesely et al. 2011). Therefore, these issues must be considered when tumor cells are used as carriers.

#### 2.1.7. Microalgae

Microalgae are unicellular microorganisms capable of photosynthesis, including eukaryotes and prokaryotes. Their cell membranes exhibit a porous structure, primarily consisting of multi-layered fibrous structures such as cellulose, proteins, and algal polysaccharides, which contribute to their large surface area, and the polysaccharides and proteins within the cell walls endow microalgae with numerous functional groups, which are characteristics that promote their potential for drug binding (Zhou et al. 2023). Microalgae also have a variety of bioactive substances, such as microalgal peptides and polysaccharides, giving them anti-tumor, anti-inflammatory, antibacterial, and antioxidant properties (Khavari et al. 2021). Certain microalgae possess unique motility, offering potential for their migration to diseased areas, for example, Spirulina can target lung tissue for drug delivery (Zhong et al. 2020). Microalgae also possess the ability to generate oxygen via photosynthesis, thereby alleviating the hypoxic conditions within tumors. This property serves as a valuable adjunct in tumor therapy, enhancing the overall efficacy of treatment (Cui et al. 2022). Unlike many other cell types, microalgae are abundant in photosynthetic pigments such as chlorophyll and exhibit fluorescence, which facilitates the tracking of drug carrier distribution and dynamics in vivo (Zhong et al. 2020). Leveraging these distinctive advantages, microalgae hold significant potential for application in the treatment of PDs.

#### 2.1.8. EVs and exosomes

EVs are membrane-bound vesicles released by cells, which can be classified into exosomes, microvesicles, and others based on their origin and size. These carriers originate from the cells themselves, and their membrane structure is similar to that of the host cells, giving them good biocompatibility and making them less likely to cause immune rejection, thereby providing a safe foundation for the treatment of PDs (Wang et al. 2022; Sharma et al. 2021; Wang et al. 2021; Elsharkasy et al. 2020). The biological barriers present in the lungs, such as alveolar epithelial cells and capillary endothelial cells, are common obstacles to drug delivery. However, these carriers, with their nanoscale size and unique membrane structure, can relatively easily cross these barriers and efficiently reach the target site. Additionally, EVs derived from lung epithelial cells, macrophages, and other sources can bind specifically to lung tissue cells via surface molecules, thereby minimizing their impact on other organs (Fujita et al. 2015). After genetic engineering modification, they can acquire controllable targeting capabilities, achieving targeted transport by binding surface molecules to target cell receptors, thereby expressing specific target ligands and further enhancing precise localization to lung lesions (Erana-Perez et al. 2024; Liang et al. 2021; Ruan et al. 2022). Furthermore, cell vesicles and exosomes can encapsulate various bioactive molecules such as proteins and nucleic acids. Their membrane structure can also protect the internal bioactive molecules from external factors such as enzymatic degradation, prolonging their circulation time in the body and ensuring effective drug delivery (Fujita et al. 2015; Weng et al. 2021). In addition, many PDs are accompanied by immune dysregulation, and stem cell-derived cell vesicles and exosomes can balance the local immune environment through immune regulation, thereby achieving the goal of improving treatment efficacy (Hu et al. 2022; Park et al. 2025).

#### 2.1.9. Cell membrane biomimetic

A biomimetic cell membrane technology is a novel drug delivery system that has emerged in recent years. It primarily utilizes the bioactive components of natural cell membranes to encapsulate drugs. Such carriers can fully retain the surface receptors and functional proteins of their source cells, enabling them to mimic the biological behavior of natural cells during in vivo delivery. This approach aims to overcome the unique physiological barriers and pathological environments of the lungs (Fang et al. 2018). Compared with traditional synthetic carriers, biomimetic cell membrane carriers have characteristics such as low immunogenicity, precise targeting, and microenvironment responsiveness (Fang et al. 2023; Tan et al. 2015). Cell membranes from different sources have different characteristics. We can select different cell membranes as carriers to deliver drugs based on different types of PDs. For example, red blood cell membranes have good biological stability and low immunogenicity, which can effectively prolong the circulation time of drugs in the body and reduce rapid drug clearance (Nguyen et al. 2023). Therefore, red blood cell membranes are widely used in the treatment of PDs; macrophage membranes can actively target the tumor microenvironment and have a natural tendency toward inflammation, making them useful in the treatment of lung cancer and PF (Wu et al. 2022; Cao et al. 2016).

### 2.2. Preparation strategies of CDDSs

**Figure 2.**
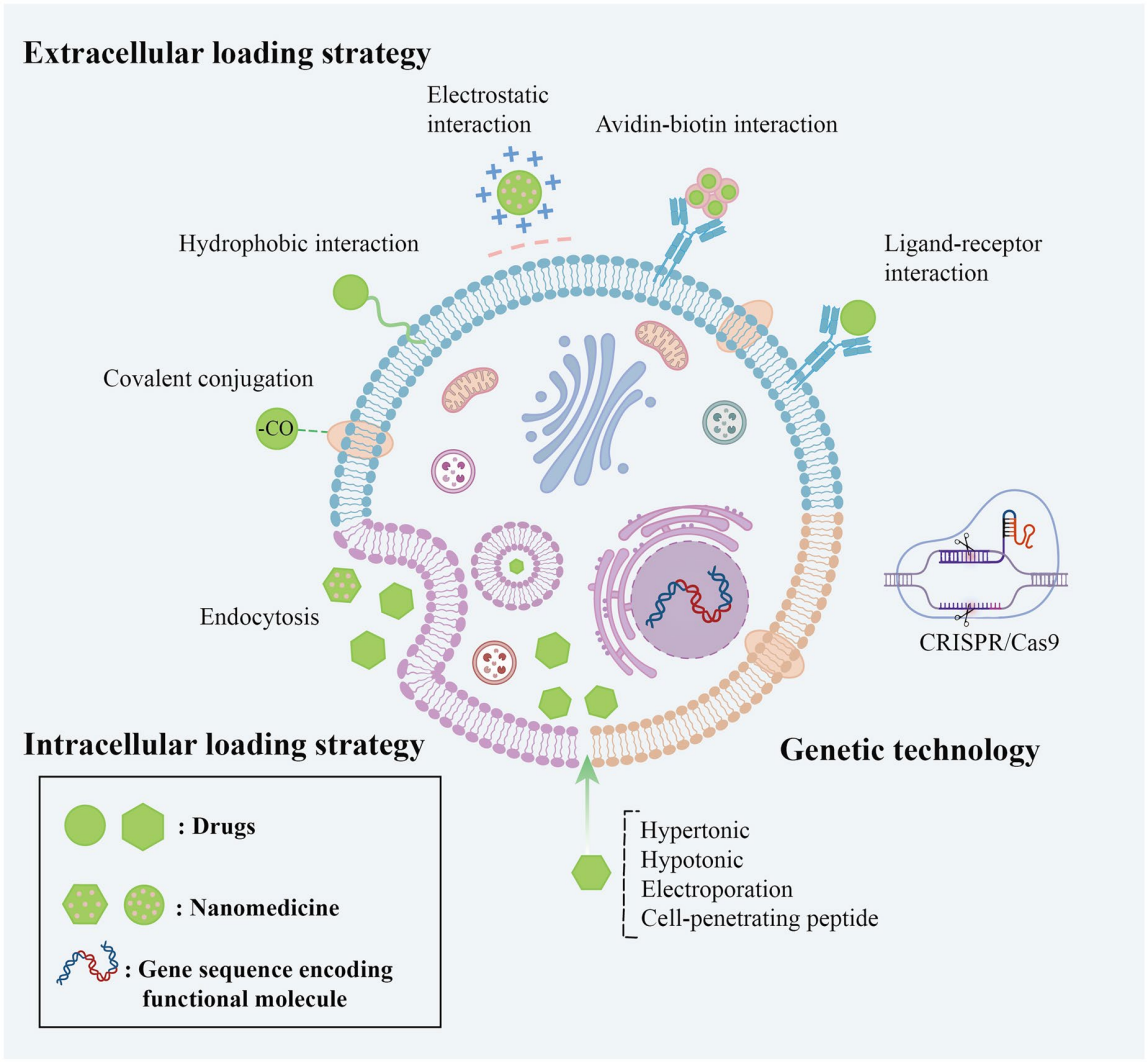
Schematic illustration of the main drug-loading strategies of CDDSs. These strategies mainly include extracellular loading strategy, intracellular loading strategy and genetic technology. The figure was illustrated by Adobe Illustrator.

Cell carriers have been prepared using a variety of techniques, such as physical adhesion, chemical modification, biological phagocytosis, and genetic engineering (Figure 2). These strategies are designed to achieve the loading of drugs or nanoparticles without demaging the cells’ natural functions. Currently, living cells can be loaded according to the characteristics of different therapeutic agents, giving full play to their biological properties in order to maximize therapeutic effects.

#### 2.2.1. Encapsulating drugs within the interior of carrier cells

The cytoplasmic compartments of cells offer ample space for drug loading, making intracellular encapsulation one of the simplest methods. Cells that naturally exhibit phagocytic abilities, such as neutrophils and macrophages, can directly ingest drugs into their cytoplasm (Guo and Qian 2024; Yuan and Hu 2024). But most cells do not possess phagocytic capabilities. For these cells, drugs are typically loaded through passive diffusion or endocytosis. These methods, however, are often constrained by the physicochemical properties of the drug, resulting in relatively low drug encapsulation efficiency. Infiltrating drugs into the cytoplasm using hypotonic solutions is a widely employed method for drug loading (Li et al. 2020). For instance, RBCs are highly permeable in hypotonic or hypertonic media and are able to contract or swell accordingly (Alshalani and Acker 2017). In a hypotonic condition, RBCs swell into a spherical shape as a result of external water entering the cell interior through osmosis. The RBC’s membrane’s surface area grows as it swells. When the membrane tension reaches a critical threshold, pores form, allowing drug molecules to pass through. The pores then seal when a hypertonic solution is added, guaranteeing that the medication molecules are securely enclosed inside the RBC (Ge et al. 2018). Another technique for drugs entry into cells is electroporation. RBCs in an isotonic solution are subjected to an electric pulse, which momentarily changes the membrane permeability and creates pores that allow drug molecules to enter the cells (Bourgeaux et al. 2016). Although this method increases the effectiveness of drug loading, it may also permanently damage the cell membrane and affect the viability or functionality of the carrier cells. Conversely, the loading technique mediated by cell-penetrating peptide (CPP) is preferable. It prevents significant harm to the erythrocyte membrane, maintains both drug activity and cellular integrity, and permits effective drug loading into erythrocytes without impairing the physiological processes of the carrier cells (He et al. 2014). However, the relatively low cytotoxicity of CPPs may still pose a risk of cellular damage (Reissmann 2014). In summary, loading drugs into living cells may cause harm to living cells, such as damage to cell membranes, loss of hemoglobin, and overall biocompatibility, which can negatively impact the delivery efficiency of living cell carriers (Li et al., 2021; Jia et al. 2025). Therefore, it is essential to consider the advantages and disadvantages of each drug loading strategy when choosing one in order to find a loading strategy that is specific to various cell types and maximizes the drug’s therapeutic efficacy.

#### 2.2.2. Attaching drugs to the surface of carrier cells

The cell membrane is made up of proteins, carbohydrates, and a phospholipid bilayer. Its surface is rich with ligands, receptors, and motifs, which allows drug carriers to connect to it (Zhang et al. 2021). An additional technique for drug loading is surface binding, which can be achieved by covalent or non-covalent interactions with the carrier cell. Covalent conjugation is usually employed to load medications onto the cell surface through chemical interactions with surface groups of cell membranes. In order to accomplish stable drug binding, this procedure frequently targets reactive groups on the cell membrane, including amino, carboxyl, and thiol groups (Glassman et al. 2021; Zhang et al. 2022). One of the reactive groups most commonly utilized for covalent conjugation is N-hydroxysuccinimide ester, which allows the drug and cell membrane to create stable amide bonds (Yang et al. 2022). Conversely, non-covalent conjugation attaches pharmaceuticals by interactions such hydrophobic, van der Waals, hydrogen bonding, and electrostatic forces. For example, by electrostatically interacting with the negatively charged cell surface, positively charged nanoparticles can stick to the surface of mammalian cells (Anselmo and Mitragotri 2014). Through π-π stacking, hydrophobic compounds like unsaturated fatty acids and aromatic amino acids can be drawn to the cell surface by the hydrophobic component of the cell membrane (de Araujo et al. 2022). Ligand-receptor interaction and avidin-biotin interaction are two other non-covalent conjugation strategies (Mu et al. 2023; Jain and Cheng 2017).

#### 2.2.3. Techniques in genetic engineering

An alternative preparation strategy involves integrating the genes encoding functional compounds into the genome of vector cells. In the field of tumor therapy, chimeric antigen receptor (CAR) technology has garnered significant attention. CAR-T cell therapy, by integrating CAR-encoding genes into the genome of T cells, enables them to specifically recognize and eliminate tumor cells (Larson and Maus 2021). Another preparation approach under this method is the direct integration of functional compound genes into the genomic DNA of vector cells, thereby achieving long-term therapeutic effects. CRISPR/Cas9, a highly efficient gene-editing tool, enables precise modification of endogenous genes across diverse cell types and species (Hryhorowicz et al. 2017). For instance, Chang et al. employed this technology to engineer human pluripotent stem cells, generating CAR-expressing neutrophils capable of targeting glioblastoma (GBM) and facilitating the specific delivery of nanomedicines (Chang et al. 2023). However, CRISPR/Cas9 carries inherent off-target risks, which may induce unintended genomic mutations, including the activation of proto-oncogenes or inactivation of tumor suppressor genes, potentially leading to adverse effects such as tumorigenesis (Tsai and Joung 2016). In addition to traditional gene editing, CRISPR-based regulatory technologies (such as CRISPRa and CRISPRi) can precisely regulate endogenous gene expression to program cells to enhance the secretion of anti-inflammatory factors or therapeutic proteins, and utilize EVs for high-purity delivery (Osteikoetxea et al. 2022). Additionally, optogenetic techniques can be used to modify MSC exosomes through spatiotemporal control, promoting wound healing in diabetes (Zhao et al. 2023); while modular RNA scaffold systems enable targeted delivery of therapeutic molecules through programmed assembly (Byrne et al. 2025). Although the application of these technologies in the field of pulmonary disease is still limited, their advantages in gene editing, targeted delivery, and dynamic regulation suggest broad potential for clinical translation.

### 2.3. Cell uptake mechanisms, endosomal escape, and drug release kinetics involved in drug delivery systems

#### 2.3.1. Cell uptake mechanisms

In live cell delivery systems, drug uptake by cells is the initial and critical step. Taking macrophages as an example, their drug uptake is primarily achieved through phagocytosis, as macrophages can directly internalize drugs or drug-loaded particles via phagocytosis (Guo and Qian 2024). In some studies, antibodies targeting tumor-associated antigens have been modified and attached to the surface of drug-loaded nanoparticles, which can enhance macrophage uptake of these particles. This approach leverages the tumor-targeting properties of macrophages to achieve drug delivery to tumor sites (Li et al. 2019). The mechanisms by which exosomes and EVs are taken up by cells include endocytosis, pinocytosis, and direct membrane fusion. Among these, endocytosis is the primary mechanism, with receptor-mediated endocytosis being the most common (Abels and Breakefield 2016). Additionally, membrane fusion is another important mechanism. The lipid bilayer of exosomes can directly fuze with the target cell membrane, releasing their contents into the cell. For example, the tetraspanin CD9, which is highly expressed in exosomes, can promote rapid membrane fusion with target cells, enabling direct cytoplasmic delivery of endogenous and exogenous therapeutic agents (Shi et al. 2020; Krylova and Feng 2023). When delivering drugs using a biomimetic cell membrane system, the cellular uptake mechanism is similar to that of natural cells or vesicles. For example, blood group glycoproteins on red blood cell membranes can interact with complementary molecules on the surface of target cells, mediating the uptake of nanoparticles. Additionally, due to the natural properties of red blood cell membranes, the risk of nanoparticles being recognized and cleared by the immune system is reduced, thereby prolonging their circulation time in the body and increasing the opportunity for contact with and uptake by target cells (Xia et al. 2019).

#### 2.3.2. Endosomal escape

Endosomal escape is a critical step in drug release. Live cell delivery systems can achieve endosomal escape through various mechanisms, including the proton sponge effect and membrane fusion mechanisms (Xu et al. 2021). Some cell-penetrating peptides (CPPs) can also be used to promote endosomal escape, helping drugs break through the endosomal membrane barrier after binding to them (Qian et al. 2016; Pei and Buyanova 2019). For exosomes and EVs, the endosomal escape mechanism involves the receptor cell internalizing EVs or exosomes, forming early endosomes that gradually mature and fuze with lysosomes to form acidified endosomes. When the pH decreases, this triggers ‘reverse fusion,’ forming fusion pores that expose and release the functional cargo within the EV lumen directly into the cytoplasm, thereby achieving transmembrane delivery (Kim et al. 2024; Hagedorn et al. 2024; Joshi et al. 2020; Zhao et al. 2024). Additionally, genetically engineering exosomes to express proteins that promote endosomal escape has emerged as a strategy to enhance exosome delivery efficiency (Nakase and Futaki 2015). The escape mechanism of biomimetic cell membranes mainly involves simulating the membrane fusion, membrane disruption, or membrane destabilization behaviors of viruses or natural cells, enabling drug- or nucleic acid-loaded nanoscale systems to escape from endosomes or lysosomes into the cytoplasm after endocytosis, thereby avoiding degradation and exerting therapeutic effects (Zhao et al. 2022).

#### 2.3.3. Drug release kinetics

The drug release kinetics of live cell delivery systems are relatively complex and influenced by multiple factors. The release of drugs within cells may be associated with cellular metabolic activity. For example, when macrophages are in an inflammatory environment, their enhanced metabolic activity may lead to changes in the membrane permeability of intracellular drug-loaded vesicles, thereby accelerating drug release (Guo and Qian 2024; Ren et al. 2021). Additionally, changes in the activity of certain enzymes within cells can also affect drug release, such as certain esterases that can hydrolyze the ester bonds on the surface of intracellular drug-loaded nanoparticles, triggering drug release (Wang et al. 2021). The drug release kinetics of exosomes and EVs are closely related to the stability of the vesicles and their interaction with target cells. Exosomes are relatively stable in the bloodstream, but once they reach the target tissue and interact with target cells, the release of their contents is triggered (Krylova and Feng 2023). Physical factors such as temperature and pH changes, as well as biological factors such as certain enzymes secreted by target cells, may affect the integrity of the exosome membrane, leading to drug release (Wang et al. 2021; Qi et al. 2016; Wang et al. 2021). For drug release in biomimetic cell membrane systems, materials responsive to stimuli such as temperature, pH, and redox conditions can be designed to induce structural changes in biomimetic nanoparticles under specific environmental conditions, thereby achieving controlled drug release (Liu et al. 2019; Kong et al. 2022).

## 3. Application advances of CDDSs in PDs

PDs seriously threaten the global health of people, and current clinical treatments for PDs are often associated with issues such as drug therapy side effects, bacterial resistance, and limited bioavailability (Zhong et al. 2021). Recent studies have demonstrated that CDDSs can significantly improve drug targeting and therapeutic efficacy in PD treatment. Consequently, CDDSs present a promising strategy for addressing PDs. This section delves into the application of living cell carriers in treating common PDs (Table 2), including lung cancer, pulmonary infections, PF, and ARDS. It also evaluate the potential of living cell carriers in enhancing drug delivery efficiency and therapeutic outcomes by analyzing the pathomechanisms of these diseases and the limitations of current therapeutic approaches.

**Table 2.** Different carriers as delivery systems in PDs.

Disease	Type of living cell-carrier	Cargo	Load technology	Reference
Lung cancer	Macrophage	Anti-HER2 chimeric antigen receptor	Genetic engineering	NCT04660929 (A Phase 1 and First in Human Study of Adenovirally 2020)
	Macrophage	Ce6	Endocytosis	(Yu et al. 2021)
	Macrophage	DOX	Electroporation	(Evangelopoulos et al. 2020)
	Platelet	Nanoparticle	Endocytosis	(Li et al., 2022)
	MSCs	Nanoparticle	Endocytosis	(Wang et al., 2019, 9)
	MSCs	Nanoparticle	Endocytosis	(Layek et al. 2018)
	MSCs	TRAIL	Genetic engineering	NCT03298763 (Targeted Stem Cells Expressing 2017)
	tumor cell	Nanoparticle	Non-covalent binding	(Liu et al., 2024)
	T cell	CAR	Genetic engineering	NCT06768151 (Clinical Study of CEA Targeting Chimeric Antigen Receptor T Lymphocytes \(CAR-T\)., 2024)
	T cell	CAR	Genetic engineering	NCT05341492 (Single-Arm 2022)
	T cell	CAR	Genetic engineering	NCT03060343 (Preliminary Clinical Study of Autologous T Cells Modified Chimeric Antigen Receptor \(CAR\)., 2017)
	T cell	CAR	Genetic engineering	NCT05274451 (A Phase 1 Study to Assess the Safety and Efficacy of LYL797797, 2022)
	T cell	CAR	Genetic engineering	NCT06006390 (Chimeric Antigen Receptor T Lymphocytes \(CAR-T\)., 2023)
	T cell	CAR	Genetic engineering	NCT03525782 (Guangzhou Anjie Biomedical Technology Co et al. 2018)
	T cell	CAR	Genetic engineering	NCT06051695 (Tempus 2023)
Pulmonary infection	Neutrophil	DBA	Endocytosis	(Chu et al. 2015)
	Neutrophil	Liposome	Endocytosis	(Li et al., 2021)
	Leucocyte	Posaconazole	Endocytosis	(Baistrocchi et al. 2017)
	Macrophage	Nanoparticle	Endocytosis	(Yue et al. 2023)
	Macrophage	Nanoparticle	Non-covalent binding	(Xu et al. 2023)
	RBCs	Liposome	Non-covalent binding	(Li et al., 2022)
	RBCs	Nanoparticle	Non-covalent binding	(Zheng et al. 2022)
	Engineered RBCs	FHA	Hypotonic dialysis	(Vizzoca et al. 2022)
	Microalgae	Nanoparticle	Covalent bonding	(Zhang et al. 2022)
	MSCs	Nanoparticle	Endocytosis	(Wang et al., 2019, 9)
PF	Monocyte	Nanoparticle	Non-covalent binding	(Chang et al. 2020)
	Macrophage	Liposome	Endocytosis	(Sang et al. 2021)
	MSCs	Let-7d/miR-154	Transduction	(Huleihel et al. 2017)
	MSCs	Liposome	Covalent bonding	(Han et al. 2023)
ARDS	RBCs	Nanoparticle	Non-covalent binding	(Sun et al. 2024)
	RBCs	Nanoparticle	Non-covalent binding	(Ding et al. 2022)
	EV	miR-21-5p	Genetic engineering	(Li et al. 2019)
	Neutrophil	Nanoparticle	Non-covalent binding	(Liu et al. 2023)
Pulmonary metastasis	Microalgae	Doxorubicin	Non-covalent binding	(Zhong et al. 2020)
	Microalgae	Nanoparticle	Covalent bonding	(Zhang et al. 2024)
	Monocyte	Nanoparticle	Endocytosis	(He et al. 2017)
	Macrophage	Salmonella	Endocytosis	(Wu et al. 2024)
	Macrophage	Nanoparticle	Endocytosis	(Wang et al. 2022)
	RBCs	Nanoparticle	Non-covalent binding	(Zhao et al. 2019)
	RBCs	Lysoma virus	Non-covalent binding	(Liu et al., 2024)
	MSCs	DOX Polymers	Endocytosis and membrane-bound	(Yao et al. 2017)

### 3.1. Lung cancer

**Figure 3.**
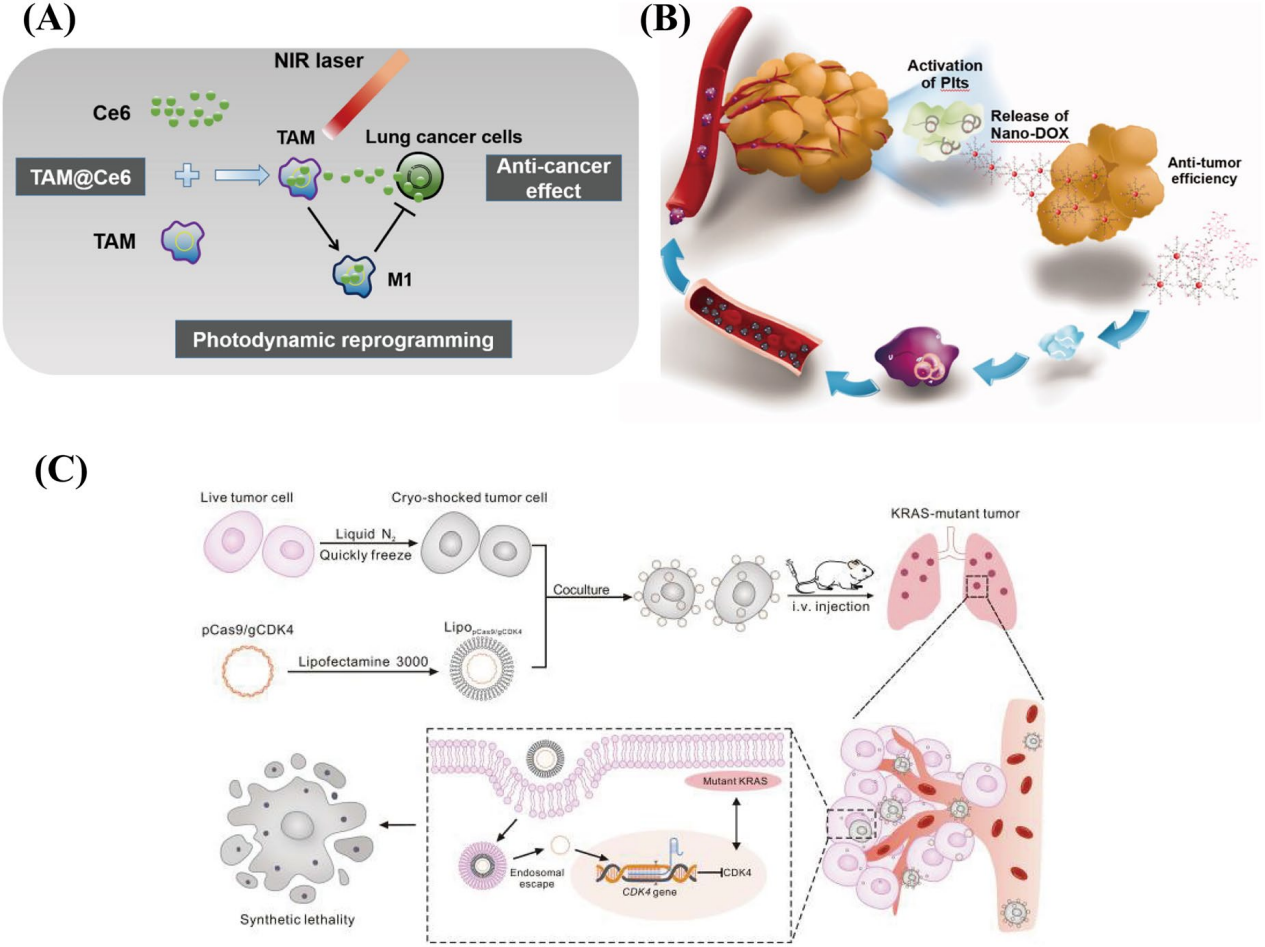
Different CDDSs in PF. (A) Ce6 delivered to lung cancer cells by macrophages causes M1 macrophage polarization through non-lethal photodynamic effects, acting as an anticancer agent. Reproduced with permission from ref. (Zhao et al. 2022). Copyright 2021, Elsevier. (B) Platelet-based drug delivery system for the treatment of lung cancer. Reproduced with permission from ref. (Ren et al. 2021). Copyright 2022, Taylor & Francis. (C) LNT cells deliver CRISPR-Cas9 nanoparticles for the treatment of KRAS-mutant NSCLC. Reproduced with permission from ref. (Kailashiya et al. 2019). Copyright 2024, Science Advances.

Globally, lung cancer is the most common malignant tumor and a serious health risk to people (Siegel et al. 2019). About 15% of it is Small Cell Lung Cancer (SCLC), while the remaining 85% is Non-Small Cell Lung Cancer (NSCLC). These two histological kinds make up the majority of it. Large cell lung cancer, lung squamous cell carcinoma, lung adenocarcinoma, and other less frequent variations are subtypes of the latter (Abdelaziz et al. 2018). Lung cancer has a terrible prognosis and a low five-year survival rate even with the availability of several treatments, including surgery, radiation therapy, chemotherapy, and targeted therapy (Li et al. 2023). Consequently, the development of novel therapeutic strategies for lung cancer is of paramount urgency. CDDSs possess the dual capabilities of targeting tumors and penetrating biological barriers, thereby facilitating the accurate delivery of drugs to the tumor site to exert therapeutic efficacy. For example, macrophages, platelets, MSCs, and tumor cells can leverage their unique biological characteristics and functions to significantly contribute to lung cancer treatment. Macrophages exhibit robust phagocytic activity and possess the capability to home in on tumor sites. These features enable them to carry and deliver drugs to lung cancer locations. Additionally, Tumor-associated macrophages (TAMs) are also important immune cells in the immunological environment around lung cancer. Capitalizing on this, Yu et al. developed a TAM-based drug delivery system to transport the photosensitizer Ce6 to lung cancer cells (Figure 3(A)). Their research showed that TAM-loaded Ce6 remained viable even when exposed to no near-infrared (NIR) light. Furthermore, within 10 hours post-NIR irradiation, the viability of TAM-loaded Ce6 remained largely unchanged, but macrophage phagocytic activity was significantly enhanced, and TAMs were successfully reprogrammed to the M1 phenotype. According to these findings, through photodynamic reprogramming, macrophages can efficiently transfer Ce6 to lung cancer cells, resulting in synergistic anti-lung cancer actions (Yu et al. 2021). Li et al. leveraged the long circulation time of platelets, their natural tendency toward tumors, and the activation-release mechanism that causes platelets to release the drug in the tumor microenvironment, and designed a CDDS in which nanodiamond-adriamycin (ND-DOX) was loaded into platelets (Figure 3(B)). Within platelets, the nanodrug showed strong stability. Experiments conducted both in vitro and in vivo further demonstrated that platelets could be stimulated to release the medication when they arrived at the tumor location, efficiently eliminating tumor cells with the least amount of harm to healthy tissues (Li et al., 2022). Both MSCs and tumor cells possess the capability to target tumor cells. Leveraging this property, researchers have developed delivery systems specifically designed for lung cancer treatment. For example, nano-engineered MSCs were prepared by introducing paclitaxel-loaded nanoparticles into MSCs through nano-engineering technology. The experimental findings revealed that in a lung cancer model, these nano-engineered MSCs could migrate to the tumor site and remain within the tumor tissue for several days, functioning as ‘cellular drug depots’ to steadily release the drug. This process effectively reduced tumor cell proliferation, suppressed tumor angiogenesis, and induced tumor cell apoptosis (Layek et al. 2018). Liu et al. employed cryo-shocked lung cancer cells as a delivery system for CRISPR-Cas9, targeting specific genes such as CDK4 to induce a synthetic lethal effect, particularly in non-small-cell lung cancers harboring KRAS mutations (Figure 3(C)). In this study, rapid liquid nitrogen shock treatment successfully eliminated the pathogenicity of lung cancer cells while preserving their structural integrity and surface receptor activity. So the lung cancer cells, whose own function was unchanged, were still able to efficiently target CRISPR-Cas9 to the tumor site in the lungs, which led to a significant down-regulation of CDK4 expression within the tumor and triggered the synthetic lethality of KRAS-mutated NSCLC that prolonged the survival of mice (Liu et al., 2024). CAR-T therapy is a novel targeted treatment that has emerged in recent years. It involves inserting the CAR gene into cells via a gene delivery carrier. Once constructed, these CAR-T cells are able to identify antigens linked to tumors (Larson and Maus 2021), CAR-T therapy holds significant promise for the treatment of lung cancer, with numerous clinical trials already underway. For example, trials are being conducted for advanced lung cancer (NCT06768151 (Clinical Study of CEA Targeting Chimeric Antigen Receptor T Lymphocytes \(CAR-T\)., 2024), NCT05341492 (Single-Arm 2022)) and non-small cell lung cancer (NCT03060343 (Preliminary Clinical Study of Autologous T Cells Modified Chimeric Antigen Receptor \(CAR\)., 2017), NCT05274451 (A Phase 1 Study to Assess the Safety and Efficacy of LYL797797, 2022)). Additionally, several clinical trials have progressed to Phase II (NCT06006390 (Chimeric Antigen Receptor T Lymphocytes \(CAR-T\)., 2023), NCT03525782 (Guangzhou Anjie Biomedical Technology Co et al. 2018), NCT06051695 (Tempus 2023)). In summary, the living cells chosen for lung cancer treatment should possess the capability to home in on the tumor site. This ensures that drugs or other therapeutic agents can effectively reach the tumor to exert anti-tumor effects while minimizing damage to normal tissues. Additionally, combination therapies, as demonstrated in the aforementioned study, can be utilized to achieve synergistic effects.

### 3.2. Lung metastasis

**Figure 4.**
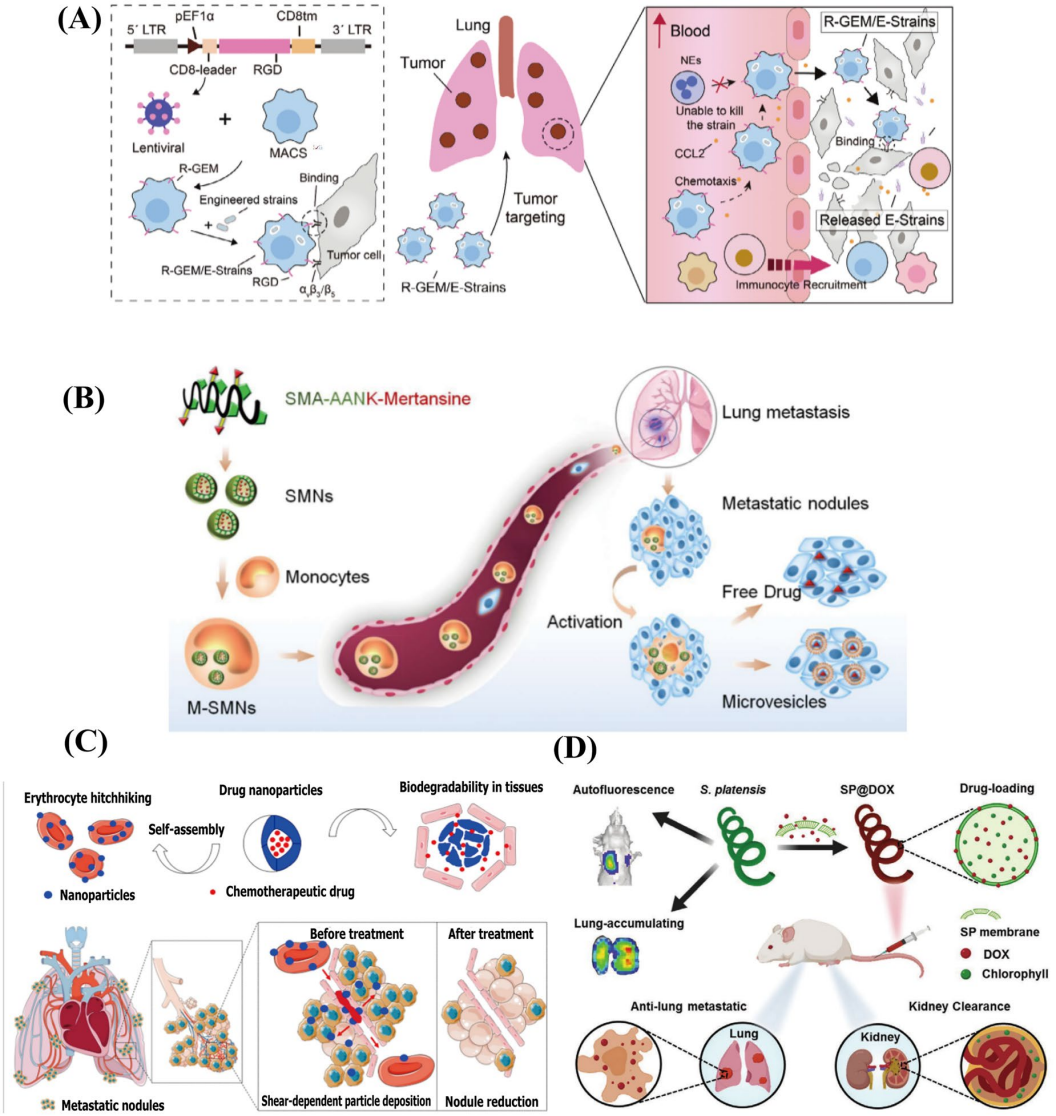
Different CDDSs in lung metastases. (A) Principles of RGD-GEM/strain cell preparation and treatment of lung metastases. Reproduced with permission from ref. (Preliminary Clinical Study of Autologous T Cells Modified Chimeric Antigen Receptor \(CAR\)., 2017). Copyright 2024, Advanced Materials. (B) Legumain-sensitive nanoparticles (M-SMNs) are transported to the pulmonary metastases of breast cancer by inflammatory monocytes. These monocytes achieve effective anti-metastatic action by facilitating medication release specific to metastases. Reproduced with permission from ref. (A Phase 1 Study to Assess the Safety and Efficacy of LYL797797, 2022). Copyright 2017, ACS. (C) The structure and therapeutic mechanism of the biodegradable drug-loaded nanoparticles assembled on the RBC - based platform (ELeCt) for targeting lung metastasis. Reproduced with permission from ref. (Guangzhou Anjie Biomedical Technology Co et al. 2018). Copyright 2019, Science Advances. (D) SP@DOX facilitates targeted drug delivery to the lungs and utilizes fluorescence imaging to guide chemotherapy, thereby inhibiting the lung metastasis of breast cancer. Reproduced with permission from ref. (Liew and Kubes 2019). Copyright 2020, Small.

Cancer metastasis is a highly lethal characteristic, defined by the spread and proliferation of cancer cells to organs distant from the primary tumor (Gerstberger et al. 2023). The lung frequently serves as a site for metastasis from various extrathoracic malignant tumors (ETMs), with the occurrence of lung metastases in patients who succumb to ETMs ranging between 20% and 54% (Francia et al. 2011; Mohammed et al. 2011). Systemic chemotherapy is a common therapeutic strategy for lung metastases (Altorki et al. 2019). However, its effectiveness has been limited due to issues with drug accumulation in the lung and inadequate targeting. CDDSs is a novel delivery system that has emerged in recent years. MSCs, macrophages, and monocytes have the ability to target tumor sites. Leveraging this targeting capability, numerous CDDSs have been designed specifically for the treatment of lung metastases. For example, a drug delivery system based on MSCs was developed through the dual drug-carrying modes of endocytosis and membrane binding. In this system, MSCs retain their stem cell properties and migratory capabilities, enabling them to remain at the lung tumor site for extended durations. As a result, they can release drugs in a targeted manner within the tumor microenvironment, thereby exerting potent anti-tumor effects. This approach not only successfully inhibits tumor growth and greatly increases the survival of tumor-bearing animals, but it also solves the problem of low drug loading in cell-derived delivery systems (Yao et al. 2017). Another study developed a novel dual-engineered microbial encapsulation therapy for macrophages (Figure 4(A)). This therapy relies on engineered macrophages, referred to as R-GEM cells, which express RGD peptides on their outer cell membrane to enhance binding to tumor cells and promote intratumoral accumulation. When co-cultured with attenuated Salmonella typhimurium VNP20009, these R-GEM cells form R-GEM/VNP cells. These cells retain bacterial bioactivity for over 24 hours and are capable of releasing anti-tumor factors to exert therapeutic effects within tumors. Furthermore, IFNγ-secreting strains (VNP-IFNγ) injected into R-GEM cells result in the formation of R-GEM/VNP-IFNγ cells. These modified cells effectively stop the growth of lung metastatic tumors in mice with colorectal, breast, and melanoma cancer models (Wu et al. 2024). He et al. developed an M-SMNs delivery system by encapsulating legumain-activated nanoparticles (SMNs) within inflammatory mononuclear cells (Figure 4(B)). This system was shown to significantly enhance drug delivery to lung metastases and achieve a penetration rate with an inhibition efficacy of 77.8% against lung metastases. This is because the SMNs remain inert within monocytes, thereby preventing premature drug release. When monocytes migrate to the tumor site and differentiate into macrophages, the high expression of legumain protease in macrophages triggers the dissociation and release of anticancer drugs from the SMNs. This mechanism effectively suppresses the proliferation, migration, and invasion of 4T1 cells (He et al. 2017). Apart from utilizing the targeted tumor site properties of the cells mentioned above, RBCs’ special physiological qualities, like their extended circulation time and biocompatibility, can be used to promote targeted drug administration and improved accumulation in the lungs. For instance, by affixing drug-loaded, biodegradable nanoparticles to the surface of RBCs, Zhao et al. created an RBC-based chemotherapy platform (Figure 4(C)). The release of nanoparticles within the lung endothelium and tumor nodules in the narrow pulmonary capillaries can be triggered by these RBCs taking advantage of the high shear stress environment once they enter the lungs. This approach achieves a tenfold increase in drug delivery to the lungs and significantly extends the circulation duration of drug-loaded nanoparticles as compared to free nanoparticles. In both early-stage and advanced lung metastasis models of melanoma, the platform has shown strong tumor growth inhibition and enhanced survival in mice (Zhao et al. 2019). The negatively charged surface of Spirulina can effectively adsorb positively charged drugs via electrostatic interactions, while the channels in its cell membrane also enable the uptake of small molecule drugs. Moreover, the chlorophyll present in Spirulina possesses natural fluorescence, which aids in tracking and monitoring its behavior in vivo. The SP@DOX formulation, prepared by loading Spirulina with adriamycin, exhibits exceptionally high drug loading efficiency and pH-responsive slow-release characteristics (Figure 4(D)). In vitro studies demonstrated that SP@DOX enhances drug release under acidic conditions, achieving cytotoxic effects comparable to those of free adriamycin while exhibiting lower toxicity in the initial phase. In vivo experiments revealed that SP@DOX rapidly accumulates in the lungs, with uptake levels significantly higher than those in other organs. In a breast cancer lung metastasis model, SP@DOX effectively inhibits lung metastasis and improves the survival rate of mice.The SP@DOX prepared by its loading with adriamycin has ultra-high drug loading efficiency and pH-responsive slow release ability (Figure 4(D)) (Zhong et al. 2020). As a result, CDDSs have shown distinct advantages in treating lung metastases. This is attributed not only to the tumor-targeting capabilities of cells such as macrophages and MSCs but also to the unique biological attributes of RBCs and the structural features of microalgae. These characteristics have collectively enhanced the potential of utilizing living cells for the treatment of lung metastases.

### 3.3. Pulmonary infection

**Figure 5.**
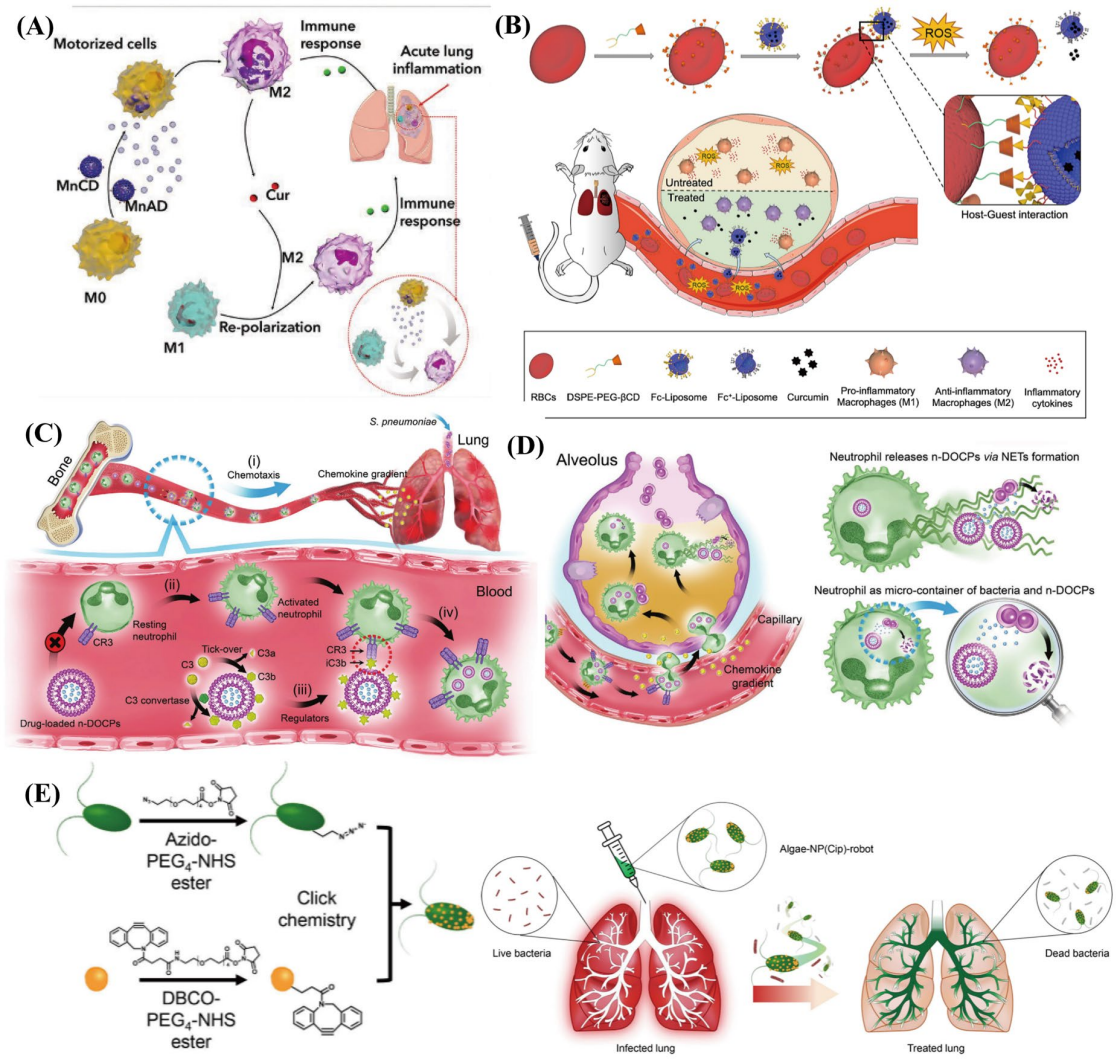
Different CDDSs in lung infection. (A) Immune modulation and macrophage polarization within the inflamed lung tissue. Reproduced with permission from ref. (Tempus 2023). Copyright 2023, Advanced Materials. (B) The inflammation-responsive supramolecular erythrocytes-hitchhiking DDS. (1) The synthesis of the RBC-NP conjugate through redox-sensitive β-CD/Fc host-guest interactions. (2) Intravenous administration of RBC-NP in mice with acute pneumonia enabled the targeted release of therapeutic agents in inflamed lung tissues, thereby effectively alleviating acute pneumonia. Reproduced with permission from ref. (Gerstberger et al. 2023). Copyright 2022, Elsevier. (C) (i) a chemokine gradient is generated in response to acute lung inflammation triggered by intranasal (i.n.) administration of S. pneumoniae. (ii) Under inflammatory conditions, neutrophils are activated and increase the expression of complement receptor 3 (CR3). (iii) Complement protein C3 is cleaved by spontaneous hydrolysis or C3 convertase to produce the activated fragment C3b, which rapidly binds to the surface of n-DOCPs and is subsequently degraded into iC3b by complement regulatory proteins. (iv) The interaction between iC3b and CR3 facilitates the uptake of n-DOCPs by activated neutrophils, rather than by resting neutrophils. Reproduced with permission from ref. (Francia et al. 2011). Copyright 2020, Advanced Materials. (D) Neutrophils loaded with n-DOCPs migrate to inflamed alveoli across the alveolar-capillary barrier driven by the chemokine gradient, and two potential mechanisms underlying the bacteria-killing effect of these neutrophils are discussed. Reproduced with permission from ref. (Francia et al. 2011). Copyright 2020, Advanced Materials. (E) The functionalization of reinhardtii with drug-loaded NP via click chemistry and the application of algae-NP robots for treating bacterial lung infections. Reproduced with permission from ref. (Mohammed et al. 2011). Copyright 2022, Nature materials.

Pulmonary infection is an infectious lesion that occurs in the interstitium or parenchyma of the lungs and is one of the diseases that affect human health. Pneumonia manifests clinically as a lung infection, primarily driven by diverse pathogens. While the advent and advancement of antibiotics and viral vaccines have significantly curtailed pneumonia-related mortality, the escalating issue of antibiotic resistance has emerged as a formidable hurdle in the clinical management of pneumonia (Zhong et al. 2021). To address this issue, various innovative antimicrobial drug delivery systems have been developed, including nanoparticle delivery systems, liposome delivery systems, and CDDSs. Among these, CDDSs provide targeted tissue delivery, extended circulation period, reduced immunogenicity, enhanced biocompatibility, and the capacity to pass through biological barriers. Leveraging these attributes, living cells can transport drugs directly to the infection site and extend the drug’s duration in the body, thereby mitigating the development of drug resistance. Yue et al. constructed a CDDS with self-propelling capabilities by assembling manganese dioxide nanoparticles inside macrophages (Figure 5(A)). They leveraged the natural tropism of macrophages toward sites of inflammation, enabling them to actively migrate toward inflamed areas in the lungs. Meanwhile, the nanoparticles catalyze the conversion of overexpressed hydrogen peroxide in the inflammatory environment to reduce inflammation and generate oxygen to propel the movement of macrophages and enhance their tissue penetration ability. In addition, curcumin-loaded nanoparticles can regulate macrophage polarization and shift them to the M2 type, which further exerts anti-inflammatory effects and effectively improves the symptoms of acute pneumonia, reduced the level of inflammatory factors, and attenuated lung edema and injury (Yue et al. 2023). In contrast, Li et al. developed an RBC-based drug delivery system by first modifying RBCs with β-cyclodextrin (β-CD) and liposomes (NPs) with ferrocene (Fc). The NPs were then attached to the RBCs through the host-guest interaction between β-CD and Fc (Figure 5(B)). Experimental results showed that in a mouse model of acute pneumonia, NPs delivered by RBCs accumulated more in the lungs of pneumonia-stricken mice than other agents. This is because reactive oxygen species (ROS) in the oxidative environment of the inflamed lung encourage the dissociation of the β-CD/Fc complex, which releases the NPs from the RBCs and permits inflammation-specific drug release. Additionally, following NP treatment, the polarization state of macrophages shifts from the pro-inflammatory M1 phenotype to the anti-inflammatory M2 phenotype, effectively reducing the inflammatory response (Li et al., 2022). This study capitalizes on the biocompatibility and long-circulating properties of RBCs, as well as the targeted drug release at the site of inflammation facilitated by host-guest chemistry, thus offering a novel therapeutic direction for pneumonia treatment. Neutrophils have the ability to target sites of inflammation and to cross biological barriers, so researchers have exploited this property to prepare liposomes composed of the inverse phosphocholine, which are able to precisely target neutrophils in the inflammation-activated state using the ‘voluntaryopsonization’ mechanism and interaction with complement receptor 3 (CR3) to precisely target neutrophils in the inflammatory activation state (Figure 5(C)). This is due to the fact that the complement fragment iC3b adsorbs to the surface of the liposome when inflammation occurs, whereas activated neutrophils have a high expression of CR3 on their surface, and the two bind specifically. In mouse models of ALI and pneumonia, neutrophils leverage their targeting and barrier-crossing abilities to migrate across the alveolar-capillary barrier and deliver liposomes to inflammatory sites in the lung. After reaching the inflammatory site, they can exert bactericidal and anti-inflammatory effects in two ways: First, by releasing drug-carrying liposomes; and second, by using neutrophils as cellular micro-containers, which contain both drug-carrying liposomes and bacteria within the cell, allowing the antibiotic to form a high concentration locally to kill the bacteria (Figure 5(D)) (Li et al., 2021). Microalgae possess the unique ability to self-propel and rapidly navigate through biofluids, which allows them to effectively distribute and penetrate deep into lung tissues. Utilizing this property, Zhang et al. designed a robotic delivery system to attach antibiotic polymer nanoparticles containing antibiotics encapsulated by neutrophil membranes to microalgae via click chemistry (Figure 5(E)). Intrathecal administration of the microrobots in an animal model led to their uniform distribution in deep lung tissues, decreased clearance of alveolar macrophages, and prolonged retention in lung tissues for over two days. These microrobots significantly reduced death rates and the bacterial burden in a mouse model of acute Pseudomonas aeruginosa pneumonia (Zhang et al. 2022). In conclusion, living cells hold significant promise for enhancing drug delivery efficiency, improving targeted drug delivery to the lungs, and minimizing systemic side effects, thus providing an alternative strategy for the treatment of lung infections.

### 3.4. PF

**Figure 6.**
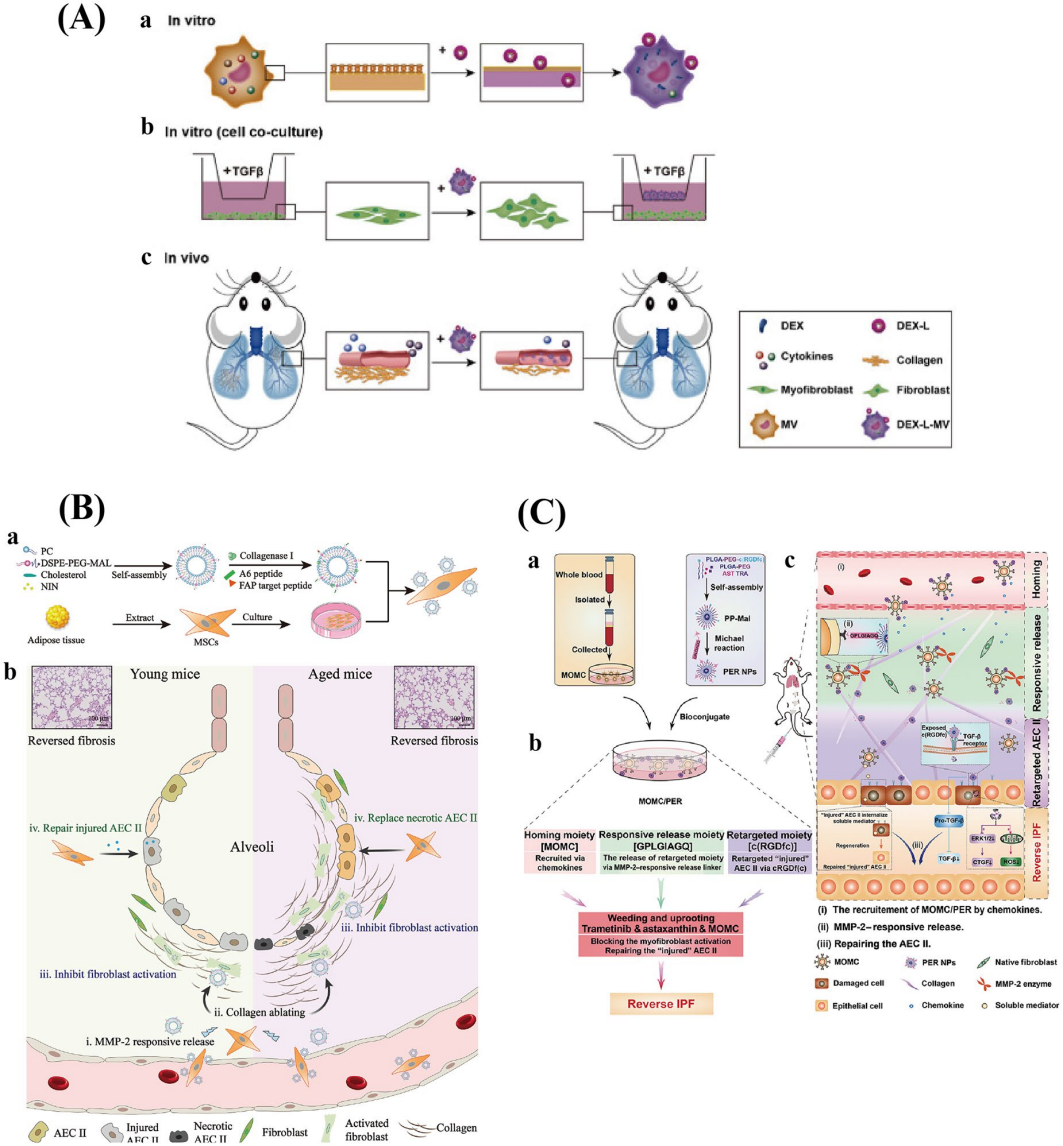
Different CDDSs in PF. (A) In vitro drug delivery targeting the macrophage-mediated inflammatory microenvironment at the cellular level (a) and in co-culture systems (b), as well as in vivo studies (c). Reproduced with permission from ref. (Zhang et al. 2022). Copyright 2021, frontiers in immunology. (B) Lung-targeting nanoengineered MSCs-Lip@NCAF was designed to reverse pulmonary fibrosis (PF) in both young and aged mice. (a) The preparation of MSCs-Lip@NCAF. (b) Due to their inherent homing ability, MSCs-Lip@NCAF migrated to injured lung tissues, where Lip@NCAF was released in response to MMP-2 activity. Subsequently, Lip@NCAF degraded collagen fibers and inhibited excessive fibroblast activation. Additionally, MSCs repaired damaged alveolar epithelial type II cells (AEC IIs) in young mice and differentiated into AEC IIs to contribute to alveolar reconstruction in aged mice. Reproduced with permission from ref. (Wynn and Barron 2010). Copyright 2023, Science advances. (C) a lung-targeted, programmed MOMC/per therapeutic system was developed to reverse IPF. (a) The bioconjugated MOMC/per was synthesized by incubating per NPs with MOMC. (b) MOMC/per incorporates multiple functional components, including a homing motif, a responsive release motif, and a retargeting motif, all aimed at reversing IPF. This system employs a ‘weeding and uprooting’ strategy to facilitate IPF reversion. (c) MOMC/per enhances drug accumulation and antifibrotic efficacy within the IPF lung microenvironment. Reproduced with permission from ref. (Han et al. 2023). Copyright 2020, Science Advances.

PF is a progressive, long-term lung disease characterized by an overabundance of extracellular matrix components, leading to lung tissue scarring and impaired respiratory performance (Wang et al. 2024). Immune dysregulation and inflammatory processes are acknowledged as major factors in the onset and advancement of PF (Zhang et al. 2018). Inflammatory cells like neutrophils and macrophages are drawn to the site of injury when the lungs are stimulated or injured (Heukels et al. 2019; Wynn and Barron 2010). Macrophages can differentiate into M1 and M2 phenotypes throughout this process (Murray 2017). M1 macrophages primarily secrete pro-inflammatory cytokines to facilitate the clearance of pathogens following alveolar epithelial damage, but they can also contribute to inflammatory injury (Shapouri-Moghaddam et al. 2018). M2 macrophages exhibit anti-inflammatory, pro-angiogenic, and tissue-repairing functions. However, over-activation of M2 macrophages can promote fibroblast proliferation and collagen production, ultimately leading to PF (Locati et al. 2013; Zhou et al. 2017). Therefore, treatment of PF can be achieved not only through pharmacological agents but also by modulating macrophage polarization. Sang et al. prepared liposomes containing dexamethasone (Dex-L), and macrophages form a macrophage delivery system (Dex-L-MV) upon uptake of Dex-L (Figure 6(A)). Dex-L has the capacity to bidirectionally modulate macrophage polarization. It can change the inflammatory microenvironment by suppressing the production of IL-6 and inducible nitric oxide synthase (iNOS) in M1 macrophages and decreasing the secretion of IL-10 and Arginase-1 (Arg-1) in M2 macrophages. In cell co-culture studies and a mouse model of PF, Dex-L-MV showed promise in reducing collagen deposition, inhibiting fibroblast migration and activation, lowering inflammatory cytokine levels in lung tissue, and lessening the severity of PF with negligible systemic toxicity (Sang et al. 2021). Furthermore, excessive ROS is released when type II alveolar epithelial cells (AECII) are damaged. This increases connective tissue growth factor (CTGF) expression and sets off the antifibrinolytic coagulation cascade. These modifications accelerate the development of PF by causing fibroblasts to become overactive and extracellular matrix (ECM) to build up (Xaubet et al. 2003; Mora et al. 2017; Guo et al. 2018). Therefore, we can start to treat PF from the repair and protection of AECII damage. Monocyte-derived multipotent cells (MOMC) proliferate and differentiate from circulating monocytes under specific conditions and possess the ability to home to injured lung tissues, where they can differentiate into various functional cells to aid in lung tissue repair. Therefore, Chang et al. took advantage of these properties of MOMC to design a delivery system that can target drugs to injured lung tissues and repair the damage of AECII (Figure 6(B)). In this study, surface-engineered nanoparticles (PERNPs) loaded with astaxanthin (AST) and trametinib (TRA) were attached to MOMC. Due to the homing ability of these cells, the PERNPs could directly reach the lungs. Upon encountering matrix metalloproteinase-2 (MMP-2) in idiopathic IPF tissues, the PERNPs would release their cargo, which could then re-target damaged AECII. The released AST enhanced the synergistic effect of TRA in inhibiting myofibroblast activation, while the MOMC facilitated the repair of damaged AECII and promoted lung regeneration (Chang et al. 2020). In contrast, Han et al. harnessed the self-renewal capacity, differentiation potential, and inflammation-targeting ability of MSCs to construct a nano-engineered platform (MSCs-Lip@NCAF) that combines MSCs with type I collagenase-modified liposome (Lip@NCAF) containing nintedanib (NIN) (Figure 6(C)). MSCs-Lip@NCAF was able to migrate to fibrotic lungs, and the released Lip @NCAF degrades collagen fibers and can deliver NIN to fibroblasts and inhibit their overactivation, while MSCs can differentiate into AECII to repair damaged alveolar structures. The in vivo experimental results demonstrated that MSCs-Lip@NCAF could improve lung function, reduce the degree of PF, and lower the level of inflammatory factors in both young and old mice without significant toxicity, and the therapeutic effect was particularly significant in old mice (Han et al. 2023). Therefore, in the treatment of PF, cells with inflammatory targeting, differentiation potential and immunomodulatory function can be selected as the delivery system, so that not only the drugs can be delivered to the lungs, but also the cells can participate in the process of immunomodulation and repair of damaged alveolar cells, and play a synergistic role, so as to achieve the role of the treatment of PF.

### 3.5. ARDS

**Figure 7.**
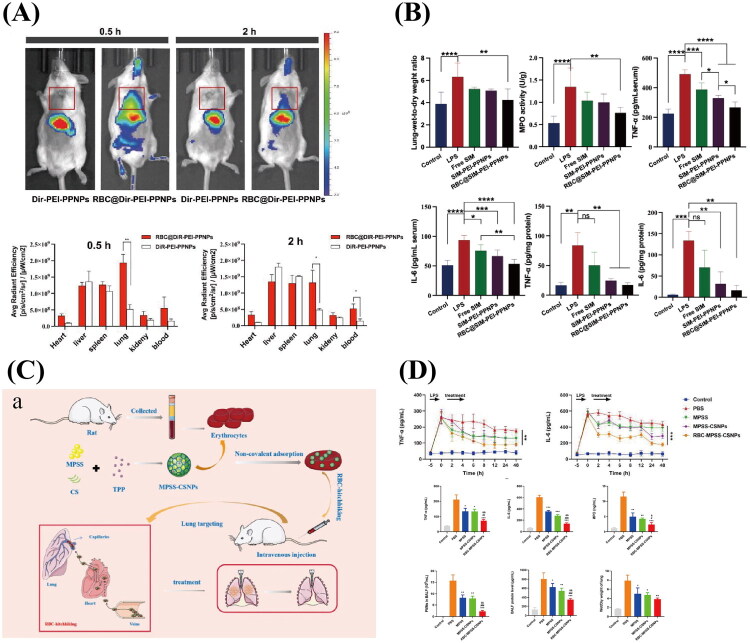
RBCs as a drug delivery system in ARDS. (A) Biodistribution of RBC@DiR-PEI-PPNPs in vivo. Reproduced with permission from ref. (Cheng et al. 2022). Copyright 2024, Int J Nanomedicine. (B) Determination of lung tissue and serum related indexes. Reproduced with permission from ref. (Cheng et al. 2022). Copyright 2024, Int J Nanomedicine. (C) Chitosan nanoparticles were prepared using the ionotropic gelation method and noncovalently attached to RBCs isolated from rat whole blood via abdominal aorta puncture. This approach enabled the efficient delivery of nanoparticles to the lungs for the treatment of inflammation. Reproduced with permission from ref. (Ren et al. 2021). Copyright 2022, Elsevier. (D) Changes in levels of inflammatory cytokines in blood and BALF in rats infected with pneumonia. Reproduced with permission from ref. (Ren et al. 2021). Copyright 2022, Elsevier.

ARDS is a clinical condition marked by widespread alveolar damage, inflammation, and edema, which can result in acute respiratory failure and compromise gas exchange and oxygenation (Battaglini et al. 2023). The pathogenesis of ARDS is highly complex, involving a range of factors, both infectious and noninfectious. These factors may either directly induce pulmonary inflammation and injury or indirectly affect the lungs by triggering systemic inflammatory responses and releasing damage-associated mediators (Bos and Ware 2022; Simou et al. 2018). Currently, the primary treatment strategies for ARDS involve respiratory support and pharmacological interventions (Fan et al. 2018). However, improper use of ventilators may exacerbate lung injury, while the unique physiological barriers of the lungs can impede effective drug delivery, resulting in reduced therapeutic efficacy and increased adverse effects (Beitler 2020; Thompson et al. 2017; Murgia et al. 2014). The ability of RBCs, platelets, neutrophils, and macrophages to gain access to lung tissue by virtue of their unique biological properties provides a novel alternative for drug delivery. For example, SIM is limited by its poor water solubility, low bioavailability, and inadequate pulmonary targeting when administered systemically for the treatment of ARDS. RBCs are ideal for drug loading because of their many beneficial properties, including their extended circulation duration, low immunogenicity, and lack of a nucleus and several organelles. Thus, a novel drug delivery system (RBC@SIM-PEI-PPNPs) was developed by adsorbing SIM-loaded nanoparticles onto the surface of RBCs via non-covalent interactions. This technique efficiently decreases adverse effects, increases drug accumulation in the lungs, and extends the duration of drug circulation (Figure 7(A)). This system’s effectiveness in treating ARDS was confirmed by both in vitro and in vivo experiments, which showed that it could significantly reduce the wet/dry weight ratio of lung tissues in ARDS mice, lower myeloperoxidase (MPO) activity, lower the levels of inflammatory factors TNF-α and IL-6 in serum and lung tissues, and improve the pathological morphology of lung tissues (Figure 7(B)) (Sun et al. 2024). Additionally, patients with ARDS are often associated with cytokine storm syndrome, leading to an excessive inflammatory response. Methylprednisolone, as a potent anti-inflammatory drug, can inhibit inflammation, but it has more side effects in the conventional administration. To address this, Ding et al. developed a drug delivery system using RBC hitchhiking chitosan nanoparticles (RBC-MPSS-CSNPs) loaded with methylprednisolone sodium succinate (MPSS), leveraging the unique properties of RBCs to prolong drug circulation and enhance pulmonary accumulation (Figure 7(C)). Experiments conducted in vivo showed that RBC-MPSS-CSNPs enhanced drug distribution in the lungs, decreased levels of inflammatory cytokines such TNF-α and IL-6 in blood and bronchoalveolar lavage fluid (BALF), and considerably prolonged the in vivo circulation time of methylprednisolone. RBC-MPSS-CSNPs also reduced inflammation and damage to lung tissue in comparison to free MPSS and MPSS-loaded chitosan nanoparticles (MPSS-CSNPs), demonstrating the system’s effectiveness in treating ARDS (Figure 7(D)) (Ding et al. 2022). Neutrophils can efficiently accumulate in the lungs and be internalized by macrophages, thereby effectively delivering drugs to inflammatory sites. This internalization process not only enhances drug targeting and local concentration but also reduces systemic side effects. Therefore, Liu et al. developed a drug delivery system based on apoptotic neutrophils by encapsulating a selective IKK-2 inhibitor in positively charged nanoparticles and loading them into apoptotic neutrophils. This delivery system can rapidly accumulate in the lungs and be internalized by macrophages via efferocytosis. Experimental results showed that T-NP@ApoNEs significantly suppressed the number of inflammatory cells in the lungs, reduced the secretion of pro-inflammatory factors, and promoted the polarization of M2 macrophages, thereby effectively improving the therapeutic outcomes of ARDS (Liu et al. 2023). Stem cells exhibit significant advantages in the treatment of ARDS, owing to their robust immunoregulatory and tissue regeneration capabilities. These properties allow stem cells to effectively promote the repair and regeneration of damaged lung tissues. Furthermore, clinical trials have already begun to evaluate the therapeutic potential of stem cell therapies in ARDS (Wilson et al. 2015; Chen et al. 2020; Hashemian et al. 2021). EVs and exosomes are characterized by high biocompatibility and low immunogenicity, enabling them to effectively avoid immune rejection reactions during in vivo delivery and reduce damage to normal tissues. This provides a safe carrier foundation for diseases such as ARDS, which are characterized by severe systemic inflammatory responses (Hu et al. 2022). Importantly, the membrane structure of exosomes is highly similar to that of the source cells, not only protecting the bioactive substances they carry from enzymatic degradation and oxidative damage, but also enabling precise targeted delivery through the binding of specific proteins on the membrane surface to receptors on the target cell surface (Fujita et al. 2015; Erana-Perez et al. 2024; Liang et al. 2021). MSC-derived exosomes can inhibit apoptosis of alveolar epithelial cells and endothelial cells by regulating related apoptotic proteins and signaling pathways, such as miR-21-5p-dependent targeting and inhibition of pro-apoptotic genes (Li et al. 2019). In addition, Li et al. designed an engineered biomimetic nanovesicle (DHA@ANeu-DDAB) by fuzing lung-targeting functional lipids, neutrophil membranes, and therapeutic lipids DHA to achieve targeted delivery to inflamed lungs and cells. DHA@ANeu-DDAB scientifically leverages the binding of β2 integrin with inflammatory cell adhesion molecules to effectively inhibit neutrophil infiltration, regulate macrophage phenotype, and promote ALI repair. This system demonstrates promising potential for targeted therapy in ARDS (Li et al. 2024). Although the use of live cell delivery systems for ARDS treatment is currently limited, EVs and exosomes have demonstrated great potential in ARDS treatment due to their high biocompatibility and low immunogenicity.

## 4. The clinical limitations and challenges of CDDSs

### 4.1. Safety and ethical issues

Living cells face some safety issues when used as carriers. For example, platelets can promote tumor angiogenesis and maintain its vascular function, as well as enhance treatment resistance by promoting cancer proliferation and forming an immunosuppressive microenvironment, while also protecting and transferring tumor cells from attack (Li et al., 2022; Zhang et al. 2020). Therefore, employing platelets as drug carriers may potentially exacerbate disease progression and deterioration. Similarly, when using RBCs as carriers, blood type compatibility must be considered, as mismatched or incompatible blood types can trigger autoimmune reactions or blood coagulation (Zhang et al. 2022). Macrophages are employed in the treatment of cancer and inflammatory diseases because of their capacity to induce therapeutic phenotypes. However, after being polarized in vitro, they can quickly respond to changes in the in vivo microenvironment and revert to a harmful pathological state (Guo and Qian 2024). Although exosomes and EVs are endogenous carriers with relatively low immunogenicity, safety concerns still exist. Their sources are complex, and if derived from diseased cells, they may carry pathogenic factors. For example, exosomes secreted by tumor cells may contain substances that promote tumor growth and metastasis, which could accelerate disease progression when used for treatment. Additionally, the separation and purification processes for exosomes and EVs have not yet been fully standardized, leading to potential variations in composition and function across different batches of products. This can result in inconsistent treatment outcomes and may also introduce impurities that trigger adverse reactions. The long-term safety of CDDSs also represents one of the major challenges currently faced. The immunogenicity of CDDSs primarily stems from cell surface antigens and damage-associated molecular patterns (DAMPs) released during the delivery process. Repeated administration disrupts the body’s immune equilibrium, triggering cumulative effects of adaptive immune responses. This leads to the production of related antibodies, resulting in rapid clearance of the delivery system and potentially inducing local inflammatory reactions (Ratanji et al. 2014). Furthermore, long-term in vitro passage of cells used to construct CDDSs may cause chromosomal instability or activation of proto-oncogenes. Upon entering the lungs, the chronic inflammatory microenvironment may further promote cellular malignant transformation. Simultaneously, therapeutic genes carried by CDDSs may induce insertion mutations, increasing carcinogenic risks. Long-term accumulation of cellular degradation products could trigger chronic granulomatous disease, while persistent disruption of pulmonary tissue architecture may lead to pulmonary fibrosis or other chronic complications. Additionally, the legitimacy of the cell source is a crucial consideration before using cells as carriers. For instance, the procurement of embryonic stem cells necessitates the destruction of human embryos, which not only sparks ethical debates but may also involve legal concerns (Bacakova et al. 2018). Induced pluripotent stem cells (iPSCs) also face ethical issues because of their unlimited differentiation capacity, and there are concerns that these cells may be used for human cloning and that their transplantation may also induce tumor formation (Aly 2020). Therefore, it is important to strictly follow ethical norms and legal guidelines when obtaining cells that involve human tissue.

### 4.2. The challenge of cell carrier delivery due to the complexity of PDs

When drugs enter the lungs, they typically need to overcome two major pulmonary barriers. The first is the mechanical barrier. When the lungs are in a diseased state, such as during an infection, it may trigger bronchial constriction or excessive mucus secretion, which can narrow the airways. Additionally, the cilia on the pulmonary mucosa can clear particles deposited in the airways, which are specific manifestations of the pulmonary mechanical barrier (Newman 2017). The second is the chemical and immune barrier, which mainly consists of surfactants, proteolytic enzymes, and alveolar macrophages (Zhong et al. 2021). Among these, pulmonary surfactant is composed of phospholipids and surfactant proteins. It covers the surface of the alveoli to form a dynamic boundary layer. The hydrophobic phospholipid layer adsorbs hydrophobic drug particles, thereby reducing the penetration efficiency of the drug (Bai et al. 2020; Cañadas et al. 2021). Proteolytic enzymes may hydrolyze certain drug carriers, leading to premature drug release (Sanders et al. 2009; Lai et al. 2009),and alveolar macrophages, as the main immune clearance cells in the lungs, recognize and phagocytose nanoparticles, thereby affecting drug efficacy (Xie et al. 2026). Artificial intelligence/machine learning (AI/ML) technology, a new technology in recent years, has shown great advantages in the treatment of pulmonary diseases (He et al. 2021). Through machine learning algorithms, large amounts of cell characteristic data and drug physicochemical property data can be analyzed to construct accurate drug-cell interaction models, thereby predicting the optimal loading conditions for specific drugs in different cell types, including loading time and drug concentration, to improve drug loading efficiency and stability. AI/ML can also integrate pulmonary physiological parameters, physicochemical properties of drug carriers, and in vitro/in vivo experimental data to construct multi-factor biodistribution prediction models. These models can precisely predict the distribution of drug carriers in different regions of the lungs during the early stages of drug development, aiding in the optimization of drug carrier design to more effectively target diseased areas while reducing distribution in non-target tissues and minimizing adverse effects. Systems Biology, Network Pharmacology (NP), and Omics-Based Modeling Technologies are particularly instrumental in optimizing CDDSs. Systems Biology enhances our understanding of drug-target interactions by constructing biological network models of diseases, thereby refining the drug release mechanisms and therapeutic efficacy of CDDSs. NP contributes to optimizing drug combinations and personalized drug design through elucidating the multi-target mechanisms of small molecules, consequently improving treatment efficiency and safety (Zhang et al. 2023). Omics-based modeling technologies—such as genomics, transcriptomics, and metabolomics—reveal disease-associated genetic variations and metabolic pathways, providing precise guidance for CDDSs design (Tran et al. 2024). Furthermore, integrating AI/ML technology with these approaches substantially enhances the therapeutic outcomes of CDDSs. Through data-driven design and predictive model refinement, these methods significantly reduce the time and cost of clinical trials, ultimately offering more precise and promising therapeutic options for PDs. However, when applying such technologies to drug development, traditional two-dimensional cell cultures and animal models exhibit significant limitations—they struggle to accurately mimic the complex physiological environment of the human lung, severely restricting the clinical translation of CDDSs. Consequently, advanced preclinical models such as lung organoids and lung organ-on-a-chip have gradually emerged as critical evaluation tools. Lung organoids, derived from stem cells, possess self-organizing capabilities to form three-dimensional structures containing multiple cell types. They highly reproduce the microscopic tissue architecture and pathological features of human lungs, providing a more pathologically relevant testing environment for evaluating the permeability, uptake efficiency, and targeting capabilities of CDDSs (Kühl et al. 2023; Purev et al. 2024). Lung organ-on-a-chip, meanwhile, dynamically simulates the mechanical conditions and air-blood barrier function during human respiration through fluid shear stress and periodic mechanical strain. It is particularly suited for studying the deposition kinetics, mucociliary clearance mechanisms, and transmembrane transport behavior of aerosolized CDDSs—key data unattainable with traditional static models (Francis et al. 2022; Zamprogno et al. 2021). Although these technologies still have room for improvement in standardization and throughput capacity, they deliver more predictive experimental data, significantly reducing uncertainties in clinical translation.

### 4.3. Production and clinical challenges

Although cells, exosomes, EVs, and biomimetic membrane delivery systems have shown promising prospects in the treatment of pulmonary diseases, there are still many challenges to their clinical application. Currently, there are no unified and comprehensive regulatory standards for cells and exosomes. The diversity of cell sources, the complex preparation process of exosomes, and the uncertainty of their composition make it difficult for regulatory agencies to establish precise and universal evaluation criteria. Take MSCsas an example: MSCs from different tissue sources exhibit differences in biological characteristics, differentiation potential, and immune regulatory functions, posing significant challenges for quality control and safety assessment. For exosomes, each step from donor cell selection, exosome separation and purification to final product quality testing lacks standardized operating procedures. Additionally, large-scale production of high-quality cells, exosomes, and related delivery systems faces numerous difficulties. Cell culture requires strict control of environmental conditions, and cell growth rates are limited, making it difficult to obtain large quantities of uniform cell populations in a short time. Similarly, the separation and purification of exosomes are difficult to scale up. While ultracentrifugation is widely used, it is time-consuming, labor-intensive, yields low output, and is prone to contamination by impurities, making it unsuitable for meeting clinical demand. Storage conditions also significantly impact the stability and activity of these delivery systems. Both cells and exosomes require low-temperature storage, but long-term cryopreservation is costly, and the freeze-thaw process can damage them, leading to reduced viability. This further complicates their clinical application. Therefore, a production protocol compliant with GMP is crucial, yet current capabilities in this area remain limited. For exosomes and engineered cells, their preparation demands even higher standards. Exosome production requires ensuring that every step is standardized and reproducible, encompassing cell culture, exosome isolation, purification, and quality control. Simultaneously, the particle size distribution, protein, and lipid composition of exosomes must undergo rigorous testing to guarantee consistency and safety. Engineered cells, meanwhile, demand rigorous cell bank management, process control, and environmental monitoring. Parameters such as culture medium composition, cell density, and temperature must be tightly controlled to guarantee consistent cell stability and differentiation potential. Although there are many challenges, the clinical translation ofCDDSs for the treatment of pulmonary diseases is accelerating. Currently, a large number of clinical trials targeting ARDS, PF, and lung cancer are underway. As one of the most clinically mature CDDSs, MSCs have been widely applied in multiple clinical trials. For example, NCT04361942: This study uses allogeneic MSCs to treat COVID-19 patients; and NCT04447833: Exploring the efficacy and safety of MSC therapy in ARDS, with related results now in the clinical evaluation phase. With advantages such as smaller size and no risk of proliferation, EVs-based therapies are receiving increasing clinical attention. These clinical trials are not only applied to the treatment of PDs (such as intravenous injection studies for COVID-19-related ARDS, NCT04493242; nebulized inhalation studies for bronchopulmonary dysplasia, NCT06919380), but also extend to the field of lung cancer diagnosis (like early detection studies, NCT03830619). Importantly, some of these trials have been completed, and preliminary results provide direct evidence of the safety and application potential of this technology. Reflecting the evolving nature of these technologies, regulatory requirements for pulmonary CDDSs are rapidly maturing. For instance, the EMA explicitly mandates aerosol characterization data for inhaled cell therapies, requiring particle size distribution profiles aligned with relevant guidelines. Similarly, FDA draft guidance on cell therapies now underscores the need for real-time imaging validation of pulmonary homing efficiency, necessitating radiometric tracking in appropriate large animal models (Cellular & Gene Therapy Guidances, FDA 2022). These specific demands highlight the heightened regulatory focus on ensuring precise delivery and function within the intricate milieu of the lung.

## 5. Conclusions and perspective

Cells, exosomes, EVs, and biomimetic membrane delivery systems are emerging carriers that bring new hope for the treatment of PDs. The inherent migration ability, barrier penetration potential, high biocompatibility, and significant drug-carrying capacity of cells show great potential in the treatment of PDs. As important mediators of intercellular communication, exosomes and EVs are rich in bioactive molecules, can regulate lung cell function, and have advantages such as low immunogenicity and good biocompatibility. They show anti-inflammatory and repair-promoting effects in the treatment of PDs. Biomimetic cell membrane delivery systems can simulate the structure and function of biological membranes, thereby enhancing the stability, targeting, and drug-carrying capacity of carriers, providing new strategies for the precise treatment of pulmonary diseases. Although they have been widely used in the treatment of pulmonary diseases, these carriers still face challenges in clinical applications. Cell carriers have limited sources, complex culture and amplification, and require long-term safety assessment. The large-scale preparation, purification, and quality control of exosomes and EVs lack standardized processes, affecting batch consistency and clinical application. The construction of biomimetic membrane delivery systems is complex, costly, and their long-term biosafety requires further investigation. In the future, it is necessary to deeply explore the mechanisms of action of these carriers, optimize preparation and delivery technologies, establish a comprehensive quality control and safety evaluation system, strengthen clinical research, and achieve clinical translation.

## Data Availability

No datasets were generated or analyzed during the current study.

## Abberivations

ALI: acute lung injury
AI/ML: Artificial intelligence/machine learning
ARDS: Acute respiratory distress syndrome
AECII: Type II alveolar epithelial cells
AST: Astaxanthin
BALF: Bronchoalveolar lavage fluid
CAR: Chimeric antigen receptor
CDDSs: Cell-derived drug delivery systems
cGAS/TLR9: cyclic GMP-AMP synthase/toll-like receptor 9
COPD: Chronic obstructive pulmonary disease
CR3: Complement receptor 3
CPP: Cell-penetrating peptide
CTGF: Connective tissue growth factor
DAMPs: Damage-associated molecular patterns
DBA: Denatured bovine serum albumin
DDSs: Drug delivery systems
ECM: Extracellular matrix
EVs: Extracellular vesicles
ESCs: Embryonic stem cells
ETM: Extrathoracic malignant tumors
Fc: Ferrocene
FPR: Formyl peptide receptor
hPSCs: Human pluripotent stem cells
iNOS: Inhibiting nitric oxide synthase
iPSC: Induced pluripotent stem cells
MOMC: Monocyte-derived pluripotent cells
MPS: Mononuclear phagocytosis system
MPSS: Methylprednisolone sodium succinate
ND-DOX: Nanodiamond-adriamycin
NETs: Neutrophil extracellular traps
NIR: Near-infrared
NP: Network Pharmacology
PNSCs: Pluripotent neural stem cells
PSCs: Pluripotent stem cells
RBCs: Red blood cells
ROS: Reactive oxygen species
SCLC: Small Cell Lung Cancer
SIM: Simvastatin
SIRPα: Signal regulatory protein α
TRA: Trametinib
β-CD: β-cyclodextrin

## References

[CIT0001] A Phase 1 Study to Assess the Safety and Efficacy of LYL797. 2022. ROR1-Targeting CAR T Cells, in Adults With Relapsed and/or Refractory Solid-Tumor Malignancies.

[CIT0002] A Phase 1, First in Human Study of Adenovirally. 2020. Transduced autologous macrophages engineered to contain an anti-her2 chimeric antigen receptor in subjects with HER2 Overexpressing solid tumors.

[CIT0003] Abdelaziz HM et al. 2018. Inhalable particulate drug delivery systems for lung cancer therapy: nanoparticles, microparticles, nanocomposites and nanoaggregates. J Control Release. 269:374–392. 10.1016/j.jconrel.2017.11.036 29180168

[CIT0004] Abels ER, Breakefield XO. 2016. Introduction to extracellular vesicles: biogenesis, RNA cargo selection, content, release, and uptake. Cell Mol Neurobiol. 36(3):301–312. 10.1007/s10571-016-0366-z 27053351 PMC5546313

[CIT0005] Agusti A, Vogelmeier CF, Halpin DMG. 2022. Tackling the global burden of lung disease through prevention and early diagnosis. Lancet Respir Med. 10(11):1013–1015. 10.1016/S2213-2600(22)00302-2 36162412

[CIT0006] Ahn J et al. 2013. Anti-tumor effect of adipose tissue derived-mesenchymal stem cells expressing interferon-β and treatment with cisplatin in a xenograft mouse model for canine melanoma. PLoS One. 8(9):e74897. 10.1371/journal.pone.0074897 24040358 PMC3767623

[CIT0007] Alshalani A, Acker JP. 2017. Red blood cell membrane water permeability increases with length of ex vivo storage. Cryobiology. 76:51–58. 10.1016/j.cryobiol.2017.04.009 28456565

[CIT0008] Altorki NK et al. 2019. The lung microenvironment: an important regulator of tumour growth and metastasis. Nat Rev Cancer. 19(1):9–31. 10.1038/s41568-018-0081-9 30532012 PMC6749995

[CIT0009] Aly RM. 2020. Current state of stem cell-based therapies: an overview. Stem Cell Investig. 7:8–8. 10.21037/sci-2020-001 PMC7367472 32695801

[CIT0010] Amaral AF, Colares PFB, Kairalla RA. 2023. Idiopathic pulmonary fibrosis: current diagnosis and treatment. J Bras Pneumol. 49(4):e20230085. 10.36416/1806-3756/e20230085 37556670 PMC10578906

[CIT0011] Anselmo AC, Mitragotri S. 2014. Cell-mediated delivery of nanoparticles: taking advantage of circulatory cells to target nanoparticles. J Control Release. 190:531–541. 10.1016/j.jconrel.2014.03.050 24747161 PMC4142097

[CIT0012] Bacakova L et al. 2018. Stem cells: their source, potency and use in regenerative therapies with focus on adipose-derived stem cells - a review. Biotechnol Adv. 36(4):1111–1126. 10.1016/j.biotechadv.2018.03.011 29563048

[CIT0013] Bai X, Li M, Hu G. 2020. Nanoparticle translocation across the lung surfactant film regulated by grafting polymers. Nanoscale. 12(6):3931–3940. 10.1039/c9nr09251j 32003385

[CIT0014] Baistrocchi SR et al. 2017. Posaconazole-loaded leukocytes as a novel treatment strategy targeting invasive pulmonary Aspergillosis. J Infect Dis. 215(11):1734–1741. 10.1093/infdis/jiw513 27799353 PMC5853238

[CIT0015] Batrakova EV, Kim MS. 2015. Using exosomes, naturally-equipped nanocarriers, for drug delivery. J Control Release. 219:396–405. 10.1016/j.jconrel.2015.07.030 26241750 PMC4656109

[CIT0016] Battaglini D et al. 2023. Challenges in ARDS definition, management, and identification of effective personalized therapies. J Clin Med. 12(4):1381. 10.3390/jcm12041381 36835919 PMC9967510

[CIT0017] Beitler JR. 2020. Lung protection in acute respiratory distress syndrome: what should we target? Curr Opin Crit Care. 26(1)34. 26- 10.1097/MCC.0000000000000692 31815776 PMC6991624

[CIT0018] Bone Marrow Mesenchymal Stem Cell Derived Extracellular Vesicles Infusion Treatment for Mild-to-Moderate COVID-19: A Phase II Clinical Trial. 2021.

[CIT0019] Bone Marrow Mesenchymal Stem Cell Derived Extracellular Vesicles for Hospitalized Patients With Moderate-to-Severe ARDS. A Phase III Clinical Trial. 2022.

[CIT0020] Bos LDJ, Ware LB. 2022. Acute respiratory distress syndrome: causes, pathophysiology, and phenotypes. Lancet. 400(10358):1145–1156. 10.1016/S0140-6736(22)01485-4 36070787

[CIT0021] Bourgeaux V, Lanao JM, Bax BE, Godfrin Y. 2016. Drug-loaded erythrocytes: on the road toward marketing approval. Drug Des Devel Ther. 10:665–676. 10.2147/DDDT.S96470 PMC4755692 26929599

[CIT0022] Byrne SM et al. 2025. An engineered U7 small nuclear RNA scaffold greatly increases ADAR-mediated programmable RNA base editing. Nat Commun. 16(1):4860. 10.1038/s41467-025-60155-z 40419487 PMC12106830

[CIT0023] Cañadas O, García-García A, Prieto MA, Pérez-Gil J. 2021. Polyhydroxyalkanoate Nanoparticles for Pulmonary Drug Delivery: interaction with Lung Surfactant. Nanomaterials (Basel). 11(6):1482. 10.3390/nano11061482 34204969 PMC8229857

[CIT0024] Cao H et al. 2016. Liposomes coated with isolated macrophage membrane can target lung metastasis of breast cancer. ACS Nano. 10(8):7738–7748. 10.1021/acsnano.6b03148 27454827

[CIT0025] Chan MH, Huang WT, Wang J, Liu RS, Hsiao M. 2020. Next-generation cancer-specific hybrid theranostic nanomaterials: MAGE-A3 NIR persistent luminescence nanoparticles conjugated to afatinib for in situ suppression of lung adenocarcinoma growth and metastasis. Adv Sci (Weinh). 7(9):1903741. 10.1002/advs.201903741 32382487 PMC7201263

[CIT0026] Chang X et al. 2020. Monocyte-derived multipotent cell delivered programmed therapeutics to reverse idiopathic pulmonary fibrosis. Sci Adv. 6(22):eaba3167. 10.1126/sciadv.aba3167 32518825 PMC7253157

[CIT0027] Chang Y et al. 2023. CAR-neutrophil mediated delivery of tumor-microenvironment responsive nanodrugs for glioblastoma chemo-immunotherapy. Nat Commun. 14(1):2266. 10.1038/s41467-023-37872-4 37080958 PMC10119091

[CIT0028] Chen J et al. 2020. Clinical study of mesenchymal stem cell treatment for acute respiratory distress syndrome induced by epidemic influenza A (H7N9) infection: a Hint for COVID-19 Treatment. Engineering (Beijing). 6(10):1153–1161. 10.1016/j.eng.2020.02.006 32292627 PMC7102606

[CIT0029] Chen J et al. 2024. DNA of neutrophil extracellular traps promote NF-κB-dependent autoimmunity via cGAS/TLR9 in chronic obstructive pulmonary disease. Signal Transduct Target Ther. 9(1):163. 10.1038/s41392-024-01881-6 38880789 PMC11180664

[CIT0030] Cheng W, Zeng Y, Wang D. 2022. Stem cell-based therapy for pulmonary fibrosis. Stem Cell Res Ther. 13(1):492. 10.1186/s13287-022-03181-8 36195893 PMC9530416

[CIT0031] Chimeric Antigen Receptor T Lymphocytes (CAR-T). 2023. Targeting CEA in the treatment of CEA positive clinical study of advanced malignant solid tumors. Shanxi Bethune, H., Ed.

[CIT0032] Chu D, Dong X, Shi X, Zhang C, Wang Z. 2018. Neutrophil-based drug delivery systems. Adv Mater. 30(22):e1706245. 10.1002/adma.201706245 29577477 PMC6161715

[CIT0033] Chu D, Gao J, Wang Z. 2015. Neutrophil-mediated delivery of therapeutic nanoparticles across blood vessel barrier for treatment of inflammation and infection. ACS Nano. 9(12):11800–11811. 10.1021/acsnano.5b05583 26516654 PMC4699556

[CIT0034] Cillóniz C, Torres A, Niederman MS. 2021. Management of pneumonia in critically ill patients. BMJ. 375:e065871. 10.1136/bmj-2021-065871 34872910

[CIT0035] Clinical Study of CEA Targeting Chimeric Antigen Receptor T Lymphocytes (CAR-T). 2024. for CEA Positive Advanced Lung Cancer.

[CIT0036] Cui H et al. 2022. How microalgae is effective in oxygen deficiency aggravated diseases? a comprehensive review of literature. Int J Nanomed. 17:3101–3122. 10.2147/IJN.S368763 PMC9297331 35874112

[CIT0037] de Araujo AD, Hoang HN, Lim J, Mak JYW, Fairlie DP. 2022. Tuning electrostatic and hydrophobic surfaces of aromatic rings to enhance membrane association and cell uptake of peptides. Angew Chem Int Ed Engl. 61(29):e202203995. 10.1002/anie.202203995 35523729 PMC9543247

[CIT0038] de Carvalho TG et al. 2023. Inhibition of murine colorectal cancer metastasis by targeting M2-TAM through STAT3/NF-kB/AKT signaling using macrophage 1-derived extracellular vesicles loaded with oxaliplatin, retinoic acid, and Libidibia ferrea. Biomed Pharmacother. 168:115663. 10.1016/j.biopha.2023.115663 37832408

[CIT0039] Della Pelle G, Kostevšek N. 2021. Nucleic acid delivery with red-blood-cell-based carriers. Int J Mol Sci. 22(10):5264. 10.3390/ijms22105264 34067699 PMC8156122

[CIT0040] Ding Y et al. 2022. RBC-hitchhiking chitosan nanoparticles loading methylprednisolone for lung-targeting delivery. J Control Release. 341:702–715. 10.1016/j.jconrel.2021.12.018 34933051 PMC8684098

[CIT0041] Dobrovolskaia MA, McNeil SE. 2007. Immunological properties of engineered nanomaterials. Nat Nanotechnol. 2(8):469–478. 10.1038/nnano.2007.223 18654343

[CIT0042] Doroudian M, MacLoughlin R, Poynton F, Prina-Mello A, Donnelly SC. 2019. Nanotechnology based therapeutics for lung disease. Thorax. 74(10):965–976. 10.1136/thoraxjnl-2019-213037 31285360

[CIT0043] Du Y, Wang S, Zhang M, Chen B, Shen Y. 2021. Cells-based drug delivery for cancer applications. Nanoscale Res Lett. 16(1):139. 10.1186/s11671-021-03588-x 34478000 PMC8417195

[CIT0044] Elsharkasy OM et al. 2020. Extracellular vesicles as drug delivery systems: why and how? Adv Drug Deliv Rev. 159:332–343. 10.1016/j.addr.2020.04.004 32305351

[CIT0045] Erana-Perez Z, Igartua M, Santos-Vizcaino E, Hernandez RM. 2024. Genetically engineered loaded extracellular vesicles for drug delivery. Trends Pharmacol Sci. 45(4):350–365. 10.1016/j.tips.2024.02.006 38508958

[CIT0046] Evangelopoulos M et al. 2020. Biomimetic cellular vectors for enhancing drug delivery to the lungs. Sci Rep. 10(1):172. 10.1038/s41598-019-55909-x 31932600 PMC6957529

[CIT0047] Fan E, Brodie D, Slutsky AS. 2018. Acute respiratory distress syndrome: advances in diagnosis and treatment. JAMA. 319(7):698–710. 10.1001/jama.2017.21907 29466596

[CIT0048] Fang RH, Gao W, Zhang L. 2023. Targeting drugs to tumours using cell membrane-coated nanoparticles. Nat Rev Clin Oncol. 20(1):33–48. 10.1038/s41571-022-00699-x 36307534

[CIT0049] Fang RH, Kroll AV, Gao W, Zhang L. 2018. Cell membrane coating nanotechnology. Adv Mater. 30(23):e1706759. 10.1002/adma.201706759 29582476 PMC5984176

[CIT0050] Ferrera MC, Labaki WW, Han MK. 2021. Advances in Chronic Obstructive Pulmonary Disease. Annu Rev Med. 72(1):119–134. 10.1146/annurev-med-080919-112707 33502902 PMC8011854

[CIT0051] Francia G, Cruz-Munoz W, Man S, Xu P, Kerbel RS. 2011. Mouse models of advanced spontaneous metastasis for experimental therapeutics. Nat Rev Cancer. 11(2):135–141. 10.1038/nrc3001 21258397 PMC4540342

[CIT0052] Francis I et al. 2022. Recent advances in lung-on-a-chip models. Drug Discov Today. 27(9):2593–2602. 10.1016/j.drudis.2022.06.004 35724916

[CIT0053] Fujita Y, Kosaka N, Araya J, Kuwano K, Ochiya T. 2015. Extracellular vesicles in lung microenvironment and pathogenesis. Trends Mol Med. 21(9):533–542. 10.1016/j.molmed.2015.07.004 26231094

[CIT0054] Ge D et al. 2018. Simulation of the osmosis-based drug encapsulation in erythrocytes. Eur Biophys J. 47(3):261–270. 10.1007/s00249-017-1255-1 28929205

[CIT0055] Geiger S, Hirsch D, Hermann FG. 2017. Cell therapy for lung disease. Eur Respir Rev. 26(144):170044. 10.1183/16000617.0044-2017 28659506 PMC9489130

[CIT0056] Gerstberger S, Jiang Q, Ganesh K. 2023. Metastasis. Cell. 186(8):1564–1579. 10.1016/j.cell.2023.03.003 37059065 PMC10511214

[CIT0057] Glassman PM et al. 2021. Red blood cells: the metamorphosis of a neglected carrier into the natural mothership for artificial nanocarriers. Adv Drug Deliv Rev. 178:113992. 10.1016/j.addr.2021.113992 34597748 PMC8556370

[CIT0058] Guangzhou Anjie Biomedical Technology Co, L.; University of Technology, S. 2018. A Clinical Study of Anti-MUC1 CAR T Cells and PD-1 Knockout Engineered T Cells for Patients With Advanced Non-small Cell Lung Cancer. Eds.

[CIT0059] Guo L et al. 2018. Interrupted reprogramming of alveolar type II cells induces progenitor-like cells that ameliorate pulmonary fibrosis. NPJ Regen Med. 3(1):14. 10.1038/s41536-018-0052-5 30210809 PMC6123410

[CIT0060] Guo M et al. 2019. Autologous tumor cell-derived microparticle-based targeted chemotherapy in lung cancer patients with malignant pleural effusion. Sci Transl Med. 11(474):eaat5690. 10.1126/scitranslmed.aat5690 30626714

[CIT0061] Guo Q, Qian ZM. 2024. Macrophage based drug delivery: key challenges and strategies. Bioact Mater. 38:55–72. 10.1016/j.bioactmat.2024.04.004 38699242 PMC11061709

[CIT0062] Hagedorn L, Jürgens DC, Merkel OM, Winkeljann B. 2024. Endosomal escape mechanisms of extracellular vesicle-based drug carriers: lessons for lipid nanoparticle design. Extracell Vesicles Circ Nucl Acids. 5(3):344–357. 10.20517/evcna.2024.19 39697635 PMC11648457

[CIT0063] Han MM et al. 2023. Nanoengineered mesenchymal stem cell therapy for pulmonary fibrosis in young and aged mice. Sci Adv. 9(29):eadg5358. 10.1126/sciadv.adg5358 37467328 PMC10355834

[CIT0064] Han X et al. 2019. Platelets as platforms for inhibition of tumor recurrence post-physical therapy by delivery of anti-PD-L1 checkpoint antibody. J Control Release. 304:233–241. 10.1016/j.jconrel.2019.05.008 31071371

[CIT0065] Han X, Li H, Zhou D, Chen Z, Gu Z. 2020. Local and targeted delivery of immune checkpoint blockade therapeutics. Acc Chem Res. 53(11):2521–2533. 10.1021/acs.accounts.0c00339 33073988 PMC8177058

[CIT0066] Han X, Wang C, Liu Z. 2018. Red blood cells as smart delivery systems. Bioconjug Chem. 29(4):852–860. 29298380 10.1021/acs.bioconjchem.7b00758

[CIT0067] Harris JC, Scully MA, Day ES. 2019. Cancer cell membrane-coated nanoparticles for cancer management. Cancers (Basel). 11(12):1836. 10.3390/cancers11121836 31766360 PMC6966582

[CIT0068] Hashemian SR et al. 2021. Mesenchymal stem cells derived from perinatal tissues for treatment of critically ill COVID-19-induced ARDS patients: a case series. Stem Cell Res Ther. 12(1):91. 10.1186/s13287-021-02165-4 33514427 PMC7844804

[CIT0069] Hassan G et al. 2019. Isolation of umbilical cord mesenchymal stem cells using human blood derivatives accompanied with explant method. Stem Cell Investig. 6:28–28. 10.21037/sci.2019.08.06 PMC6789207 31620475

[CIT0070] He H et al. 2014. Cell-penetrating peptides meditated encapsulation of protein therapeutics into intact red blood cells and its application. J Control Release. 176:123–132. 10.1016/j.jconrel.2013.12.019 24374002 PMC3939723

[CIT0071] He S, Leanse LG, Feng Y. 2021. Artificial intelligence and machine learning assisted drug delivery for effective treatment of infectious diseases. Adv Drug Deliv Rev. 178:113922. 10.1016/j.addr.2021.113922 34461198

[CIT0072] He X et al. 2017. Inflammatory monocytes loading protease-sensitive nanoparticles enable lung metastasis targeting and intelligent drug release for anti-metastasis therapy. Nano Lett. 17(9):5546–5554. 10.1021/acs.nanolett.7b02330 28758755

[CIT0073] He Z, Zhang Y, Feng N. 2020. Cell membrane-coated nanosized active targeted drug delivery systems homing to tumor cells: a review. Mater Sci Eng C Mater Biol Appl. 106:110298. 31753336 10.1016/j.msec.2019.110298

[CIT0074] Heukels P, Moor CC, von der Thüsen JH, Wijsenbeek MS, Kool M. 2019. Inflammation and immunity in IPF pathogenesis and treatment. Respir Med. 147:79–91. 10.1016/j.rmed.2018.12.015 30704705

[CIT0075] Hryhorowicz M, Lipiński D, Zeyland J, Słomski R. 2017. CRISPR/Cas9 immune system as a tool for genome engineering. Arch Immunol Ther Exp (Warsz). 65(3):233–240. 10.1007/s00005-016-0427-5 27699445 PMC5434172

[CIT0076] Hu Q et al. 2022. Extracellular vesicles in the pathogenesis and treatment of acute lung injury. Mil Med Res. 9(1):61. 10.1186/s40779-022-00417-9 36316787 PMC9623953

[CIT0077] Huleihel L et al. 2017. Modified mesenchymal stem cells using miRNA transduction alter lung injury in a bleomycin model. Am J Physiol Lung Cell Mol Physiol. 313(1):L92–l103. 28385811 10.1152/ajplung.00323.2016 PMC5538868

[CIT0078] Jain A, Cheng K. 2017. The principles and applications of avidin-based nanoparticles in drug delivery and diagnosis. J Control Release. 245:27–40. 27865853 10.1016/j.jconrel.2016.11.016 PMC5222781

[CIT0079] Jia B et al. 2025. Engineering of Erythrocytes as Drug Carriers for Therapeutic Applications. Adv Biol (Weinh). 9(5):e2400242. 10.1002/adbi.202400242 39037400

[CIT0080] Jiang A et al. 2018. Doxorubicin-loaded silicon nanoparticles impregnated into red blood cells featuring bright fluorescence, strong photostability, and lengthened blood residency. Nano Res. 11(4):2285–2294. 10.1007/s12274-017-1850-6

[CIT0081] Joshi BS, de Beer MA, Giepmans BNG, Zuhorn IS. 2020. Endocytosis of extracellular vesicles and release of their cargo from endosomes. ACS Nano. 14(4):4444–4455. 10.1021/acsnano.9b10033 32282185 PMC7199215

[CIT0082] Kailashiya J, Gupta V, Dash D. 2019. Engineered human platelet-derived microparticles as natural vectors for targeted drug delivery. Oncotarget. 10(56):5835–5846. 10.18632/oncotarget.27223 31645903 PMC6791386

[CIT0083] Khavari F, Saidijam M, Taheri M, Nouri F. 2021. Microalgae: therapeutic potentials and applications. Mol Biol Rep. 48(5):4757–4765. 10.1007/s11033-021-06422-w 34028654 PMC8142882

[CIT0084] Khayambashi P et al. 2021. Hydrogel encapsulation of mesenchymal stem cells and their derived exosomes for tissue engineering. Int J Mol Sci. 22(2):684. 10.3390/ijms22020684 33445616 PMC7827932

[CIT0085] Kim HI et al. 2024. Recent advances in extracellular vesicles for therapeutic cargo delivery. Exp Mol Med. 56(4):836–849. 10.1038/s12276-024-01201-6 38556545 PMC11059217

[CIT0086] Koleva L, Bovt E, Ataullakhanov F, Sinauridze E. 2020. Erythrocytes as carriers: from drug delivery to biosensors. Pharmaceutics. 12(3):276. 10.3390/pharmaceutics12030276 32197542 PMC7151026

[CIT0087] Kolios G, Moodley Y. 2013. Introduction to stem cells and regenerative medicine. Respiration. 85(1):3–10. 10.1159/000345615 23257690

[CIT0088] Kong F et al. 2022. A biomimetic nanocomposite with enzyme-like activities and CXCR4 antagonism efficiently enhances the therapeutic efficacy of acute myeloid leukemia. Bioact Mater. 18:526–538. 35415298 10.1016/j.bioactmat.2022.03.022 PMC8976099

[CIT0089] Krylova SV, Feng D. 2023. The machinery of exosomes: biogenesis, release, and uptake. Int J Mol Sci. 24(2):1337. 10.3390/ijms24021337 36674857 PMC9865891

[CIT0090] Kühl L et al. 2023. Human LUNG ORGANOIDS-A NOVEL EXPERIMENTAL AND PRECISION MEDICINE APPROACH. Cells. 12(16):2067. 10.3390/cells12162067 37626876 PMC10453737

[CIT0091] Lai SK, Wang YY, Hanes J. 2009. Mucus-penetrating nanoparticles for drug and gene delivery to mucosal tissues. Adv Drug Deliv Rev. 61(2):158–171. 10.1016/j.addr.2008.11.002 19133304 PMC2667119

[CIT0092] Landesman-Milo D, Ramishetti S, Peer D. 2015. Nanomedicine as an emerging platform for metastatic lung cancer therapy. Cancer Metastasis Rev. 34(2):291–301. 10.1007/s10555-015-9554-4 25948376

[CIT0093] Larson RC, Maus MV. 2021. Recent advances and discoveries in the mechanisms and functions of CAR T cells. Nat Rev Cancer. 21(3):145–161. 10.1038/s41568-020-00323-z 33483715 PMC8353572

[CIT0094] Layek B, Sadhukha T, Panyam J, Prabha S. 2018. Nano-engineered mesenchymal stem cells increase therapeutic efficacy of anticancer drug through true active tumor targeting. Mol Cancer Ther. 17(6):1196–1206. 10.1158/1535-7163.MCT-17-0682 29592881 PMC5984697

[CIT0095] Lei X et al. 2020. Immune cells within the tumor microenvironment: biological functions and roles in cancer immunotherapy. Cancer Lett. 470:126–133. 10.1016/j.canlet.2019.11.009 31730903

[CIT0096] Li CX et al. 2019. Artificially reprogrammed macrophages as tumor-tropic immunosuppression-resistant biologics to realize therapeutics production and immune activation. Adv Mater. 31(15):e1807211. 10.1002/adma.201807211 30803083

[CIT0097] Li J et al. 2022. Supramolecular erythrocytes-hitchhiking drug delivery system for specific therapy of acute pneumonia. J Control Release. 350:777–786. 10.1016/j.jconrel.2022.08.029 35995300

[CIT0098] Li JW, Wei L, Han Z, Chen Z. 2019. Mesenchymal stromal cells-derived exosomes alleviate ischemia/reperfusion injury in mouse lung by transporting anti-apoptotic miR-21-5p. Eur J Pharmacol. 852:68–76. 30682335 10.1016/j.ejphar.2019.01.022

[CIT0099] Li QR et al. 2022. Platelets are highly efficient and efficacious carriers for tumor-targeted nano-drug delivery. Drug Deliv. 29(1):937–949. 10.1080/10717544.2022.2053762 35319321 PMC8956315

[CIT0100] Li S et al. 2021. Voluntary-opsonization-enabled precision nanomedicines for inflammation treatment. Adv Mater. 33(3):e2006160. 10.1002/adma.202006160 33296121

[CIT0101] Li W, Su Z, Hao M, Ju C, Zhang C. 2020. Cytopharmaceuticals: an emerging paradigm for drug delivery. J Control Release. 328:313–324. 10.1016/j.jconrel.2020.08.063 32889055

[CIT0102] Li X et al. 2024. Engineered biomimetic nanovesicles based on neutrophils for hierarchical targeting therapy of acute respiratory distress syndrome. ACS Nano. 18(2):1658–1677. 10.1021/acsnano.3c09848 38166370

[CIT0103] Li Y et al. 2021. Clinical progress and advanced research of red blood cells based drug delivery system. Biomaterials. 279:121202. 10.1016/j.biomaterials.2021.121202 34749072

[CIT0104] Li Y, Yan B, He S. 2023. Advances and challenges in the treatment of lung cancer. Biomed Pharmacother. 169:115891. 10.1016/j.biopha.2023.115891 37979378

[CIT0105] Li YJ et al. 2021. Artificial exosomes for translational nanomedicine. J Nanobiotechnology. 19(1):242. 10.1186/s12951-021-00986-2 34384440 PMC8359033

[CIT0106] Liang T et al. 2021. Recent Advances in Macrophage-Mediated Drug Delivery Systems. Int J Nanomedicine. 16:2703–2714. 10.2147/IJN.S298159 33854316 PMC8039204

[CIT0107] Liang Y, Duan L, Lu J, Xia J. 2021. Engineering exosomes for targeted drug delivery. Theranostics. 11(7):3183–3195. 10.7150/thno.52570 33537081 PMC7847680

[CIT0108] Liew PX, Kubes P. 2019. The neutrophil’s role during health and disease. Physiol Rev. 99(2):1223–1248. 10.1152/physrev.00012.2018 30758246

[CIT0109] Liu F et al. 2024. Cryo-shocked tumor cells deliver CRISPR-Cas9 for lung cancer regression by synthetic lethality. Sci Adv. 10(13):eadk8264. 10.1126/sciadv.adk8264 38552011 PMC10980270

[CIT0110] Liu G et al. 2019. Engineering biomimetic platesomes for pH-responsive drug delivery and enhanced antitumor activity. Adv Mater. 31(32):e1900795. 10.1002/adma.201900795 31222856

[CIT0111] Liu M et al. 2024. Erythrocyte-leveraged oncolytic virotherapy (ELeOVt): oncolytic virus assembly on erythrocyte surface to combat pulmonary metastasis and alleviate side effects. Adv Sci (Weinh). 11(5):e2303907. 10.1002/advs.202303907 37997186 PMC10837356

[CIT0112] Liu W et al. 2024. Theoretical basis, state and challenges of living cell-based drug delivery systems. Theranostics. 14(13):5152–5183. 10.7150/thno.99257 39267776 PMC11388066

[CIT0113] Liu X et al. 2023. Apoptotic neutrophil-mediated inflammatory microenvironment regulation for the treatment of ARDS. Nano Today. 52:101946. 10.1016/j.nantod.2023.101946

[CIT0114] Locati M, Mantovani A, Sica A. 2013. Macrophage activation and polarization as an adaptive component of innate immunity. Adv Immunol. 120:163–184. 24070384 10.1016/B978-0-12-417028-5.00006-5

[CIT0115] Lu Y, Hu Q, Jiang C, Gu Z. 2019. Platelet for drug delivery. Curr Opin Biotechnol. 58:81–91. 10.1016/j.copbio.2018.11.010 30529814

[CIT0116] Mesenchymal Stromal Cells for the Treatment of Moderate to Severe COVID-19. 2020. Acute respiratory distress syndrome. Mesoblast, I.; National Heart, L.; Blood, I., Eds.

[CIT0117] Mohammed TL et al. 2011. ACR appropriateness criteria^®^ screening for pulmonary metastases. J Thorac Imaging. 26(1):W1–3. 10.1097/RTI.0b013e3182010bf9 21258219

[CIT0118] Mora AL, Rojas M, Pardo A, Selman M. 2017. Emerging therapies for idiopathic pulmonary fibrosis, a progressive age-related disease. Nat Rev Drug Discov. 16(11):755–772. 10.1038/nrd.2017.170 28983101

[CIT0119] Mu Q et al. 2023. Ligustrazine nanoparticle hitchhiking on neutrophils for enhanced therapy of cerebral ischemia-reperfusion injury. Adv Sci (Weinh). 10(19):e2301348. 10.1002/advs.202301348 37078794 PMC10323616

[CIT0120] Mukherjee D, Bhatt S. 2022. Biocomposite-based nanostructured delivery systems for the treatment and control of inflammatory lung diseases. Nanomedicine (Lond). 17(12):845–863. 10.2217/nnm-2021-0425 35477308

[CIT0121] Murgia X, de Souza Carvalho C, Lehr C-M. 2014. Overcoming the pulmonary barrier: new insights to improve the efficiency of inhaled therapeutics. European Journal of Nanomedicine. 6(3):157–169. 10.1515/ejnm-2014-0019

[CIT0122] Murray PJ. 2017. Macrophage Polarization. Annu Rev Physiol. 79:541–566. 10.1146/annurev-physiol-022516-034339 27813830

[CIT0123] Nakase I, Futaki S. 2015. Combined treatment with a pH-sensitive fusogenic peptide and cationic lipids achieves enhanced cytosolic delivery of exosomes. Sci Rep. 5(1):10112. 10.1038/srep10112 26011176 PMC4443764

[CIT0124] Newman SP. 2017. Drug delivery to the lungs: challenges and opportunities. Ther Deliv. 8(8):647–661. 28730933 10.4155/tde-2017-0037

[CIT0125] Nguyen PHD, Jayasinghe MK, Le AH, Peng B, Le MT. 2023. Advances in drug delivery systems based on red blood cells and their membrane-derived nanoparticles. ACS Nano. 17(6):5187–5210. 10.1021/acsnano.2c11965 36896898

[CIT0126] Olsson M, Bruhns P, Frazier WA, Ravetch JV, Oldenborg PA. 2005. Platelet homeostasis is regulated by platelet expression of CD47 under normal conditions and in passive immune thrombocytopenia. Blood. 105(9):3577–3582. 10.1182/blood-2004-08-2980 15665111 PMC1895021

[CIT0127] Olsson M, Nilsson A, Oldenborg PA. 2006. Target cell CD47 regulates macrophage activation and erythrophagocytosis. Transfus Clin Biol. 13(1-2):39–43. 10.1016/j.tracli.2006.02.013 16564725

[CIT0128] Osteikoetxea X et al. 2022. Engineered Cas9 extracellular vesicles as a novel gene editing tool. J Extracell Vesicles. 11(5):e12225. 10.1002/jev2.12225 35585651 PMC9117459

[CIT0129] Park KS, Lässer C, Lötvall J. 2025. Extracellular vesicles and the lung: from disease pathogenesis to biomarkers and treatments. Physiol Rev. 105(3):1733–1821. 10.1152/physrev.00032.2024 40125970

[CIT0130] Pei D, Buyanova M. 2019. Overcoming endosomal entrapment in drug delivery. Bioconjug Chem. 30(2):273–283. 10.1021/acs.bioconjchem.8b00778 30525488 PMC6501178

[CIT0131] Preliminary Clinical Study of Autologous T Cells Modified Chimeric Antigen Receptor (CAR). Hunan Yongren Medical Innovation Co, L. 2017. Targeting PD-L1 and CD80/CD86 (zeushield cytotoxic T lymphocytes) for the treatment of recurrent or refractory non small cell lung cancer. Hunan Zhaotai Yongren Medical Innovation Co, L.; Eds.

[CIT0132] Purev E, Bahmed K, Kosmider B. 2024. Alveolar organoids in lung disease modeling. Biomolecules. 14(1):115. 10.3390/biom14010115 38254715 PMC10813493

[CIT0133] Qi H et al. 2016. Blood exosomes endowed with magnetic and targeting properties for cancer therapy. ACS Nano. 10(3):3323–3333. 10.1021/acsnano.5b06939 26938862

[CIT0134] Qian Z et al. 2016. Discovery and mechanism of highly efficient cyclic cell-penetrating peptides. Biochemistry. 55(18):2601–2612. 10.1021/acs.biochem.6b00226 27089101 PMC8562596

[CIT0135] Rabe DC et al. 2021. Tumor extracellular vesicles regulate macrophage-driven metastasis through CCL5. Cancers (Basel). 13(14):3459. 10.3390/cancers13143459 34298673 PMC8303898

[CIT0136] Ratanji KD, Derrick JP, Dearman RJ, Kimber I. 2014. Immunogenicity of therapeutic proteins: influence of aggregation. J Immunotoxicol. 11(2):99–109. 10.3109/1547691X.2013.821564 23919460 PMC4002659

[CIT0137] Reissmann S. 2014. Cell penetration: scope and limitations by the application of cell-penetrating peptides. J Pept Sci. 20(10):760–784. 10.1002/psc.2672 25112216

[CIT0138] Ren K et al. 2021. Macrophage-mediated multi-mode drug release system for photothermal combined with anti-inflammatory therapy against postoperative recurrence of triple negative breast cancer. Int J Pharm. 607:120975. 10.1016/j.ijpharm.2021.120975 34363913

[CIT0139] Rezaie J, Nejati V, Mahmoodi M, Ahmadi M. 2022. Mesenchymal stem cells derived extracellular vesicles: a promising nanomedicine for drug delivery system. Biochem Pharmacol. 203:115167. 10.1016/j.bcp.2022.115167 35820499

[CIT0140] Rong R et al. 2022. Blood cell-based drug delivery systems: a biomimetic platform for antibacterial therapy. Eur J Pharm Biopharm. 177:273–288. 10.1016/j.ejpb.2022.07.009 35868489

[CIT0141] Ruan S et al. 2022. Extracellular vesicles as an advanced delivery biomaterial for precision cancer immunotherapy. Adv Healthc Mater. 11(5):e2100650. 34197051 10.1002/adhm.202100650 PMC8720116

[CIT0142] Sanders N, Rudolph C, Braeckmans K, De Smedt SC, Demeester J. 2009. Extracellular barriers in respiratory gene therapy. Adv Drug Deliv Rev. 61(2):115–127. 10.1016/j.addr.2008.09.011 19146894 PMC7103358

[CIT0143] Sang X et al. 2021. Macrophage-targeted lung delivery of dexamethasone improves pulmonary fibrosis therapy via Regulating the immune microenvironment. Front Immunol. 12:613907. 10.3389/fimmu.2021.613907 33679754 PMC7935565

[CIT0144] Sellheyer K, Krahl D. 2010. Skin mesenchymal stem cells: prospects for clinical dermatology. J Am Acad Dermatol. 63(5):859–865. 10.1016/j.jaad.2009.09.022 20471137

[CIT0145] Shapouri-Moghaddam A et al. 2018. Macrophage plasticity, polarization, and function in health and disease. J Cell Physiol. 233(9):6425–6440. 10.1002/jcp.26429 PMC13482560 29319160

[CIT0146] Sharma S et al. 2021. Extracellular vesicle nanoarchitectonics for novel drug delivery applications. Small. 17(42):e2102220. 10.1002/smll.202102220 34216426

[CIT0147] Shi X et al. 2020. Genetically engineered cell-derived nanoparticles for targeted breast cancer immunotherapy. Mol Ther. 28(2):536–547. 10.1016/j.ymthe.2019.11.020 31843452 PMC7001084

[CIT0148] Siegel RL, Miller KD, Jemal A. 2019. Cancer statistics, 2019. CA Cancer J Clin. 69(1):7–34. 10.3322/caac.21551 30620402

[CIT0149] Simou E, Leonardi-Bee J, Britton J. 2018. The effect of alcohol consumption on the risk of ards: a systematic review and meta-analysis. Chest. 154(1):58–68. 10.1016/j.chest.2017.11.041 29288645 PMC6045784

[CIT0150] Single-Arm A. 2022. Open, exploratory clinical study evaluating the safety and efficacy of EGFR/B7H3 CAR-T in patients with EGFR/B7H3-positive advanced solid tumors (lung and triplenegative breast cancer).

[CIT0151] Su Y, Zhang T, Huang T, Gao J. 2021. Current advances and challenges of mesenchymal stem cells-based drug delivery system and their improvements. Int J Pharm. 600:120477. 10.1016/j.ijpharm.2021.120477 33737099

[CIT0152] Sun M et al. 2024. Red blood cell-hitchhiking delivery of simvastatin to relieve acute respiratory distress syndrome. Int J Nanomedicine. 19:5317–5333. 38859953 10.2147/IJN.S460890 PMC11164090

[CIT0153] Tan S, Wu T, Zhang D, Zhang Z. 2015. Cell or cell membrane-based drug delivery systems. Theranostics. 5(8):863–881. 10.7150/thno.11852 26000058 PMC4440443

[CIT0154] Targeted Stem Cells Expressing. TRAIL as a therapy for lung cancer. 2017.

[CIT0155] Tempus, A. I., 2023. A seamless phase 1/2 study to evaluate the safety and efficacy of A2B694, an autologous logic-gated Tmod™ CAR T, in heterozygous HLA-A*02 adults with recurrent unresectable, locally advanced, or metastatic solid tumors that express MSLN and have lost HLA-A*02 expression.

[CIT0156] Thomas BL et al. 2024. Molecular determinants of neutrophil extracellular vesicles that drive cartilage regeneration in inflammatory arthritis. Arthritis Rheumatol. 76(12):1705–1718. 10.1002/art.42958 39041647 PMC11605269

[CIT0157] Thompson BT, Chambers RC, Liu KD. 2017. Acute Respiratory Distress Syndrome. N Engl J Med. 377(6):562–572. 10.1056/NEJMra1608077 28792873

[CIT0158] Thu HE, Haider M, Khan S, Sohail M, Hussain Z. 2023. Nanotoxicity induced by nanomaterials: a review of factors affecting nanotoxicity and possible adaptations. OpenNano. 14:100190. 10.1016/j.onano.2023.100190

[CIT0159] Tran TO, Vo TH, Le NQK. 2024. Omics-based deep learning approaches for lung cancer decision-making and therapeutics development. Brief Funct Genomics. 23(3):181–192. 10.1093/bfgp/elad031 37519050

[CIT0160] Tsai SQ, Joung JK. 2016. Defining and improving the genome-wide specificities of CRISPR-Cas9 nucleases. Nat Rev Genet. 17(5):300–312. 10.1038/nrg.2016.28 27087594 PMC7225572

[CIT0161] Uccelli A, Moretta L, Pistoia V. 2008. Mesenchymal stem cells in health and disease. Nat Rev Immunol. 8(9):726–736. 10.1038/nri2395 19172693

[CIT0162] Vesely MD, Kershaw MH, Schreiber RD, Smyth MJ. 2011. Natural innate and adaptive immunity to cancer. Annu Rev Immunol. 29(1):235–271. 10.1146/annurev-immunol-031210-101324 21219185

[CIT0163] Vijayan V, Uthaman S, Park IK. 2018. Cell membrane-camouflaged nanoparticles: a promising biomimetic strategy for cancer theragnostics. Polymers (Basel). 10(9):983. 10.3390/polym10090983 30960908 PMC6404000

[CIT0164] Villa CH et al. 2015. Delivery of drugs bound to erythrocytes: new avenues for an old intravascular carrier. Ther Deliv. 6(7):795–826. 10.4155/tde.15.34 26228773 PMC4712023

[CIT0165] Vizzoca A et al. 2022. Erythro-magneto-HA-virosome: a bio-inspired drug delivery system for active targeting of drugs in the lungs. Int J Mol Sci. 23(17):9893. 10.3390/ijms23179893 36077300 PMC9455992

[CIT0166] Wan Q et al. 2023. Inhaled nano-based therapeutics for pulmonary fibrosis: recent advances and future prospects. J Nanobiotechnology. 21(1):215. 10.1186/s12951-023-01971-7 37422665 PMC10329312

[CIT0167] Wang BZ, Luo LJ, Vunjak-Novakovic G. 2022. RNA and protein delivery by cell-secreted and bioengineered extracellular vesicles. Adv Healthc Mater. 11(5):e2101557. 10.1002/adhm.202101557 34706168 PMC8891029

[CIT0168] Wang H, Zang J, Zhao Z, Zhang Q, Chen S. 2021. The advances of neutrophil-derived effective drug delivery systems: a key review of managing tumors and inflammation. Int J Nanomedicine. 16:7663–7681. 10.2147/IJN.S328705 34815670 PMC8605828

[CIT0169] Wang J et al. 2021. Designer exosomes enabling tumor targeted efficient chemo/gene/photothermal therapy. Biomaterials. 276:121056. 10.1016/j.biomaterials.2021.121056 34364178

[CIT0170] Wang J et al. 2024. Pulmonary fibrosis: pathogenesis and therapeutic strategies. MedComm (2020). 5(10):e744. 10.1002/mco2.744 39314887 PMC11417429

[CIT0171] Wang J, Chen D, Ho EA. 2021. Challenges in the development and establishment of exosome-based drug delivery systems. J Control Release. 329:894–906. 10.1016/j.jconrel.2020.10.020 33058934

[CIT0172] Wang LT et al. 2016. Human mesenchymal stem cells (MSCs) for treatment towards immune- and inflammation-mediated diseases: review of current clinical trials. J Biomed Sci. 23(1):76. 10.1186/s12929-016-0289-5 27809910 PMC5095977

[CIT0173] Wang X et al. 2019.Efficient lung cancer-targeted drug delivery via a nanoparticle/MSC system. Acta Pharm Sin B. 9(1):167–176. 30766788 10.1016/j.apsb.2018.08.006 PMC6362298

[CIT0174] Wang Y et al. 2021. Tuning the efficacy of esterase-activatable prodrug nanoparticles for the treatment of colorectal malignancies. Biomaterials. 270:120705. 10.1016/j.biomaterials.2021.120705 33581609

[CIT0175] Wang Y et al. 2022. Nanotherapeutic macrophage-based immunotherapy for the peritoneal carcinomatosis of lung cancer. Nanoscale. 14(6):2304–2315. 10.1039/d1nr06518a 35083479

[CIT0176] Wei X et al. 2013. Mesenchymal stem cells: a new trend for cell therapy. Acta Pharmacol Sin. 34(6):747–754. 10.1038/aps.2013.50 23736003 PMC4002895

[CIT0177] Weng Z et al. 2021. Therapeutic roles of mesenchymal stem cell-derived extracellular vesicles in cancer. J Hematol Oncol. 14(1):136. 10.1186/s13045-021-01141-y 34479611 PMC8414028

[CIT0178] Wilson JG et al. 2015. Mesenchymal stem (stromal) cells for treatment of ARDS: a phase 1 clinical trial. Lancet Respir Med. 3(1):24–32. 10.1016/S2213-2600(14)70291-7 25529339 PMC4297579

[CIT0179] Wróblewska A, Szczygieł A, Szermer-Olearnik B, Pajtasz-Piasecka E. 2023. Macrophages as promising carriers for nanoparticle delivery in anticancer therapy. Int J Nanomed. 18:4521–4539. 10.2147/IJN.S421173 PMC10422973 37576466

[CIT0180] Wu L et al. 2024. Dual-Engineered Macrophage-Microbe Encapsulation for Metastasis Immunotherapy. Adv Mater. 36(36):e2406140. 10.1002/adma.202406140 39023382

[CIT0181] Wu Y et al. 2022. Macrophage cell membrane-based nanoparticles: a new promising biomimetic platform for targeted delivery and treatment. J Nanobiotechnology. 20(1):542. 10.1186/s12951-022-01746-6 36575429 PMC9794113

[CIT0182] Wynn TA, Barron L. 2010. Macrophages: master regulators of inflammation and fibrosis. Semin Liver Dis. 30(3):245–257. 10.1055/s-0030-1255354 20665377 PMC2924662

[CIT0183] Xaubet A et al. 2003. Transforming growth factor-beta1 gene polymorphisms are associated with disease progression in idiopathic pulmonary fibrosis. Am J Respir Crit Care Med. 168(4):431–435. 10.1164/rccm.200210-1165OC 12746254

[CIT0184] Xia Q, Zhang Y, Li Z, Hou X, Feng N. 2019. Red blood cell membrane-camouflaged nanoparticles: a novel drug delivery system for antitumor application. Acta Pharm Sin B. 9(4):675–689. 10.1016/j.apsb.2019.01.011 31384529 PMC6663920

[CIT0185] Xiao G et al. 2022. Platelets for cancer treatment and drug delivery. Clin Transl Oncol. 24(7):1231–1237. 10.1007/s12094-021-02771-x 35218523

[CIT0186] Xie M, Tao L, Zhang Z, Wang W. 2021. Mesenchymal stem cells mediated drug delivery in tumor-targeted therapy. Curr Drug Deliv. 18(7):876–891. 10.2174/1567201817999200819140912 32819256

[CIT0187] Xie S, Fatemipayam N, Zhang J, Aichele CP, Ramsey JD. 2026. Inhalable micro/nanoparticles for pulmonary delivery of macromolecular therapeutics: A review. Biomaterials. 325:123550. 10.1016/j.biomaterials.2025.123550 40680709

[CIT0188] Xu E, Saltzman WM, Piotrowski-Daspit AS. 2021. Escaping the endosome: assessing cellular trafficking mechanisms of non-viral vehicles. J Control Release. 335:465–480. 10.1016/j.jconrel.2021.05.038 34077782

[CIT0189] Xu W et al. 2024. Safe induction of acute inflammation with enhanced antitumor immunity by hydrogel-mediated outer membrane vesicle delivery. Small Methods. 8(10):e2301620. 10.1002/smtd.202301620 38343178

[CIT0190] Xu X et al. 2019. Tumor cells modified with newcastle disease virus expressing IL-24 as a cancer vaccine. Mol Ther Oncolytics. 14:213–221. 10.1016/j.omto.2019.06.001 31338417 PMC6630061

[CIT0191] Xu X, Kwong CHT, Li J, Wei J, Wang R. 2023. “Zombie” macrophages for targeted drug delivery to treat acute pneumonia. ACS Appl Mater Interfaces. 15(24):29012–29022. 10.1021/acsami.3c06025 37291057

[CIT0192] Yang L, Yang Y, Chen Y, Xu Y, Peng J. 2022. Cell-based drug delivery systems and their in vivo fate. Adv Drug Deliv Rev. 187:114394. 10.1016/j.addr.2022.114394 35718252

[CIT0193] Yao S et al. 2017. Maximized nanodrug-loaded mesenchymal stem cells by a dual drug-loaded mode for the systemic treatment of metastatic lung cancer. Drug Deliv. 24(1):1372–1383. 28920712 10.1080/10717544.2017.1375580 PMC8241180

[CIT0194] Yu TT et al. 2021. Macrophages mediated delivery of chlorin e6 and treatment of lung cancer by photodynamic reprogramming. Int Immunopharmacol. 100:108164. 10.1016/j.intimp.2021.108164 34562845

[CIT0195] Yuan S, Hu Q. 2024. Convergence of nanomedicine and neutrophils for drug delivery. Bioact Mater. 35:150–166. 10.1016/j.bioactmat.2024.01.022 38318228 PMC10839777

[CIT0196] Yue L et al. 2023. Chemotaxis-guided self-propelled macrophage motor for targeted treatment of acute pneumonia. Adv Mater. 35(20):e2211626. 10.1002/adma.202211626 36905923

[CIT0197] Zamprogno P et al. 2021. Second-generation lung-on-a-chip with an array of stretchable alveoli made with a biological membrane. Commun Biol. 4(1):168. 10.1038/s42003-021-01695-0 33547387 PMC7864995

[CIT0198] Zhang E, Phan P, Algarni HA, Zhao Z. 2022. Red Blood cell inspired strategies for drug delivery: emerging concepts and new advances. Pharm Res. 39(11):2673–2698. 10.1007/s11095-022-03328-5 35794397

[CIT0199] Zhang F et al. 2022. Nanoparticle-modified microrobots for in vivo antibiotic delivery to treat acute bacterial pneumonia. Nat Mater. 21(11):1324–1332. 10.1038/s41563-022-01360-9 36138145 PMC9633541

[CIT0200] Zhang F et al. 2024. Biohybrid microrobots locally and actively deliver drug-loaded nanoparticles to inhibit the progression of lung metastasis. Sci Adv. 10(24):eadn6157. 10.1126/sciadv.adn6157 38865468 PMC11168470

[CIT0201] Zhang L et al. 2018. Macrophages: friend or foe in idiopathic pulmonary fibrosis? Respir Res. 19(1):170. 10.1186/s12931-018-0864-2 30189872 PMC6127991

[CIT0202] Zhang P et al. 2023. Network pharmacology: towards the artificial intelligence-based precision traditional Chinese medicine. Brief Bioinform. 25(1):1–12. 10.1093/bib/bbad518 PMC10777171 38197310

[CIT0203] Zhang SY, Zhou ZR, Qian RC. 2021. Recent progress and perspectives on cell surface modification. Chem Asian J. 16(21):3250–3258. 10.1002/asia.202100852 34427996

[CIT0204] Zhang Y et al. 2020. Platelet-specific PDGFB Ablation Impairs Tumor Vessel Integrity and Promotes Metastasis. Cancer Res. 80(16):3345–3358. 10.1158/0008-5472.CAN-19-3533 32586981

[CIT0205] Zhao J et al. 2024. Unignored intracellular journey and biomedical applications of extracellular vesicles. Adv Drug Deliv Rev. 212:115388. 10.1016/j.addr.2024.115388 38969268

[CIT0206] Zhao X et al. 2022. Herpesvirus-mimicking DNAzyme-loaded nanoparticles as a mitochondrial DNA stress inducer to activate innate immunity for tumor therapy. Adv Mater. 34(37):e2204585. 10.1002/adma.202204585 35869026

[CIT0207] Zhao X et al. 2023. Optogenetic engineered umbilical cord MSC-derived exosomes for remodeling of the immune microenvironment in diabetic wounds and the promotion of tissue repair. J Nanobiotechnology. 21(1):176. 10.1186/s12951-023-01886-3 37269014 PMC10236791

[CIT0208] Zhao Z, Ukidve A, Gao Y, Kim J, Mitragotri S. 2019. Erythrocyte leveraged chemotherapy (ELeCt): nanoparticle assembly on erythrocyte surface to combat lung metastasis. Sci Adv. 5(11):eaax9250. 10.1126/sciadv.aax9250 31763454 PMC6853768

[CIT0209] Zheng J et al. 2022. Red blood cell-hitchhiking mediated pulmonary delivery of ivermectin: effects of nanoparticle properties. Int J Pharm. 619:121719. 10.1016/j.ijpharm.2022.121719 35390488 PMC8978457

[CIT0210] Zhong D, Li W, Qi Y, He J, Zhou M. 2020. Photosynthetic biohybrid nanoswimmers system to alleviate tumor hypoxia for FL/PA/MR imaging-guided enhanced radio-photodynamic synergetic therapy. Adv Funct Materials. 30(17):1910395. 10.1002/adfm.201910395

[CIT0211] Zhong D, Zhang D, Xie T, Zhou M. 2020. Biodegradable microalgae-based carriers for targeted delivery and imaging-guided therapy toward lung metastasis of breast cancer. Small. 16(20):e2000819. 10.1002/smll.202000819 32297465

[CIT0212] Zhong W, Zhang X, Zeng Y, Lin D, Wu J. 2021. Recent applications and strategies in nanotechnology for lung diseases. Nano Res. 14(7):2067–2089. 10.1007/s12274-020-3180-3 33456721 PMC7796694

[CIT0213] Zhou D et al. 2017. Macrophage polarization and function: new prospects for fibrotic disease. Immunol Cell Biol. 95(10):864–869. 10.1038/icb.2017.64 29044201

[CIT0214] Zhou M et al. 2023. Applications of microalga-powered microrobots in targeted drug delivery. Biomater Sci. 11(23):7512–7530. 10.1039/d3bm01095c 37877241

[CIT0215] Zhu Y et al. 2024. Platelet-derived drug delivery systems: pioneering treatment for cancer, cardiovascular diseases, infectious diseases, and beyond. Biomaterials. 306:122478. 10.1016/j.biomaterials.2024.122478 38266348

